# Factors driving effective population size and pan-genome evolution in bacteria

**DOI:** 10.1186/s12862-018-1272-4

**Published:** 2018-10-12

**Authors:** Louis-Marie Bobay, Howard Ochman

**Affiliations:** 1 Department of Integrative Biology, University of Texas at Austin, Austin, TX 78712 USA; 2 Department of Biology, University of North Carolina at Greensboro, 321 McIver Street, PO Box 26170, Greensboro, NC 27402 USA

**Keywords:** Effective population size, Gene repertoires, Genome architecture, Drift, Prokaryotes

## Abstract

**Background:**

Knowledge of population-level processes is essential to understanding the efficacy of selection operating within a species. However, attempts at estimating effective population sizes (*Ne*) are particularly challenging in bacteria due to their extremely large census populations sizes, varying rates of recombination and arbitrary species boundaries.

**Results:**

In this study, we estimated *Ne* for 153 species (152 bacteria and one archaeon) defined under a common framework and found that ecological lifestyle and growth rate were major predictors of *Ne*; and that contrary to theoretical expectations, *Ne* was unaffected by recombination rate. Additionally, we found that *Ne* shapes the evolution and diversity of total gene repertoires of prokaryotic species.

**Conclusion:**

Together, these results point to a new model of genome architecture evolution in prokaryotes, in which pan-genome sizes, not individual genome sizes, are governed by drift-barrier evolution.

**Electronic supplementary material:**

The online version of this article (10.1186/s12862-018-1272-4) contains supplementary material, which is available to authorized users.

## Background

Population dynamics dictate the evolution of species, such that organisms with large effective population sizes (*Ne*) evolve under effective selection, preventing most deleterious alleles to reach fixation in the population, and those with small *Ne* are more susceptible to genetic drift, whereby alleles can sometimes reach fixation irrespective of their adaptive value. Like other traits, the structure of genomes is shaped by selection and drift, such that organisms with smaller *Ne* accumulate weakly deleterious sequences, such as mobile elements, intergenic DNA, and introns [1]. Conversely, in species with large *Ne*, deleterious sequences have a low probability of reaching fixation through stochastic processes and are eliminated by selection. Thus, the genomes of species with large *Ne* would be expected to lack slightly deleterious, non-functional sequences, and the genomes of species with small population sizes would possess such sequences [1, 2]. For these reasons, *Ne* is thought to be the main parameter driving the evolution of genome size in eukaryotes and in bacteria [1–3].

Multiple parameters contribute to differences in *Ne* across organisms. Naturally, census population size and its fluctuation over time are the primary determinants of *Ne*. Population substructure can reduce *Ne* through non-random breeding in sexual species, such that *Ne* is animals is largely governed by parental investment and fecundity rather than geographic range or demographic perturbations [4]. In contrast, the determinants of *Ne* remain largely enigmatic for microbial organisms. Whereas microbes often reach enormous census population sizes, estimates of their effective populations sizes are usually many orders of magnitude lower [5]. This discrepancy between predicted and observed population sizes suggests that demographic fluctuations and other mechanisms contribute to the loss of a large part of their genetic diversity.

Estimating the effective population sizes of bacterial species has been considered problematic for several reasons: (*i*) Genomic-based methods used to estimate *Ne* rely on segregating alleles at neutral sites, but since selection might potentially be acting on every nucleotide position in bacterial genomes [6], identification of strictly neutral sites is challenging. Moreover, the imprint of selection is a time-dependent process [7], so *Ne* estimates that consider any non-neutral sites must be adjusted for divergence time. (*ii*) Due to clonality and genomic linkage, both background selection against deleterious alleles and selective sweeps of beneficial alleles result in the loss of polymorphism. These processes, better known as Hill-Robertson effects [8], are thought to strongly impair most common estimators of *Ne* in asexual or variably recombining organisms [9]. (*iii*) *Ne* estimates depend on the population in question—typically entire species—and the delineation of species boundaries in bacteria has been fraught with difficulties [10].

In this study, we apply a standardized framework that uniformly defines species borders to derive relative and absolute estimates of *Ne* across Bacteria and Archaea. We examine multiple traits that can potentially affect *Ne* across a set of 153 prokaryotic species, and the relationship between *Ne* and genome size and pan-genome size. By further analyzing the relationship between drift and population size on the complete gene repertoires of bacterial species, we show that pan-genome size—rather than absolute genome size—is likely shaped by the effectiveness of selection across species.

## Results

### Variation of *Ne* across bacterial phyla and lifestyles

We based our estimates of effective population size on two methods: *dN/dS*, which estimates the effectiveness of selection and was used as a proxy for *Ne* in 153 species, and Watterson’s estimator, which was applied to those 10 species whose mutation rates are available [11, 12]. To ensure that comparisons of *Ne* were robust across taxa, we (*i*) defined species based on a uniform set of criteria, (*ii*) computed *dN/dS* ratios on a common set of universally distributed genes, and (*iii*) limited analyses to a specific sequence-divergence interval. Both methods for estimating effective population size yielded similar values and indicated that *Ne* of most bacterial species is on the order of 10^8^–10^9^. (Additional file 1: Table S1). Five species displayed much lower *Ne* (*Aggregatibacter actinomycetemcomitans*, *Bordetella pertussis*, *Tropheryma whipplei* and *Yersinia pestis*), and of all species considered, *Mycoplasma pneumoniae* had the smallest effective population size (*Ne* = 3.8 × 10^6^). Similar values were obtained for *Ne* when computed from the entire core genome of each species or from the 44 universally distributed genes (Additional file 1: Table S1 and Additional file 2: Figure S1).

**Fig. 1:**
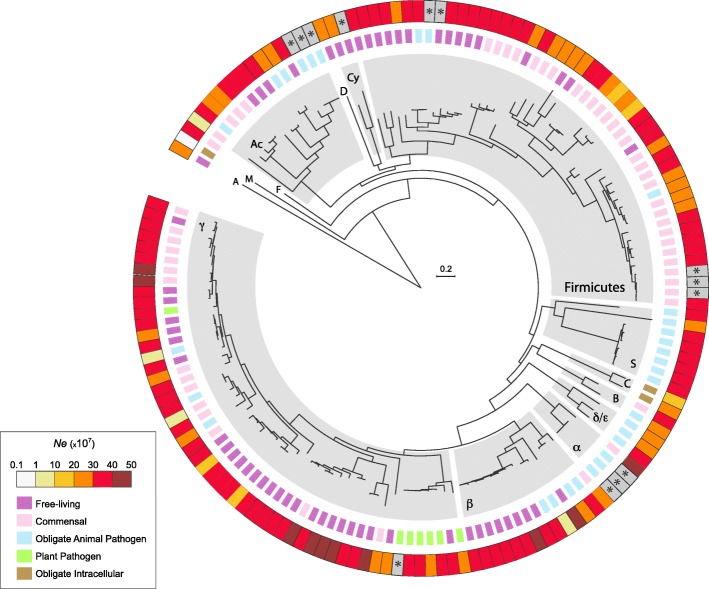
Variation in effective population size (*Ne*) across bacteria. The effective population size of each bacterial species is estimated from the average *dS/dN*, normalized across species by considering a common set of universally distributed genes (outer circle) for strains within a species whose divergence at synonymous sites were in the range of 0.1 ≤ *dS* ≤ 0.3. Grey shading denotes those species that do not contain strains within the considered divergence range of *dS* values. Bacterial lifestyles are indicated in the inner circle. The maximum likelihood tree was built on the concatenate of prokaryote-universal proteins with one representative strain for each species. The scale bar indicates the substitution rate per site. The major prokaryote clades are indicated in the tree (clockwise): A: *Archaea*, M: *Mycoplasma*, F: *Fusobacteria*, Ac: *Actinobacteria*, D: *Dehalococcoides*, Cy: *Cyanobacteria/Melainabacteria*, S: *Spirochaetia*, C: *Chlamydiae*, B: *Bacteroidetes/Chlorobi*, δ/ε: *Delta- and Epsilonproteobacteria*, α: *Alphaproteobacteria*, β: *Betaproteobacteria*, γ: *Gammaproteobacteria*

**Fig. 2:**
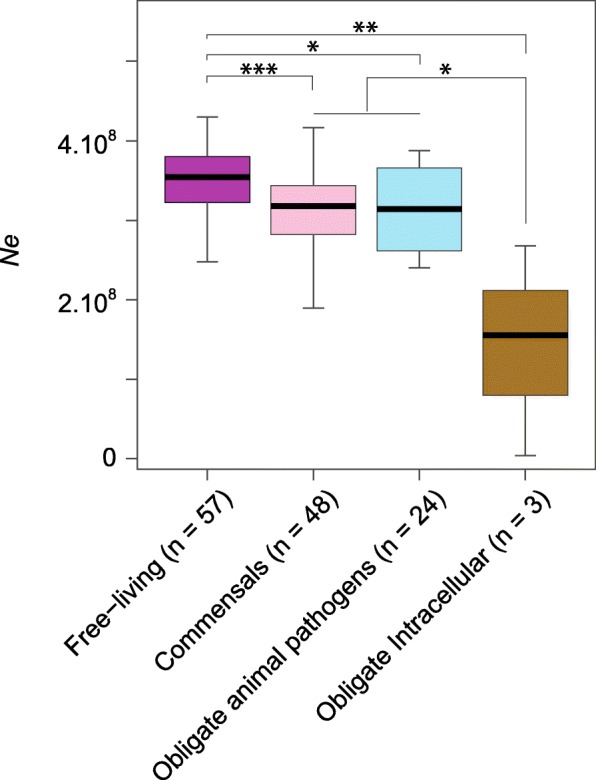
Correlation between bacterial lifestyle and effective population size. *Ne* values and lifestyles follow those presented in Fig. 1, with the number of species in each lifestyle category indicated (need to change the location of numbers to under lifestyle category). Plant pathogens were not represented, since it was often unclear whether they were obligate or facultative pathogens. ***P < 0.001, **P < 0.01, *P < 0.05, Wilcoxon test

We tested the impact of phylogeny on *Ne*, testing whether more closely related lineages yielded more similar estimates of *Ne*. We built the phylogenetic tree of the 153 analyzed species (Fig. 1) and correlated phylogenetic distances (see Methods) with the dissimilarity in effective population sizes, defined for each species pair as *|Ne*_*i*_ *- Ne*_*j*_*|* for species *i* and *j*, respectively. As evidenced by the high scatter of points and low correlation coefficient (Additional file 3: Figure S2), *Ne* is weakly but significantly predicted by the phylogenetic relationship of the different species (Spearman’s rho = 0.17, *P* < 10^− 15^). More closely related species tend to exhibit more similar values of *Ne*; however, closely related species often share similar lifestyles (Fig. 1), and there is a very strong association between species lifestyles and *Ne* (Figs. 1 and 2). For clarity, we present results as *dS/dN* values—instead of the customary *dN/dS* values—because it scales positively with *Ne*, and in each figure, the expected *Ne* values are extrapolated from the *dS/dN* metrics. As expected, free-living bacteria display the largest *Ne*, and obligate endosymbiotic bacteria the smallest (Fig. 2 and Additional file 1: Table S1), while commensals and obligate pathogens have intermediate values. Similar results were obtained when *dS/dN* ratios were based on the entire core genome of each species as when limited to the set of universally distributed genes (Additional file 1: Table S1 and Additional file 4: Figure S3). Within each of the lifestyle categories, there is variation of *Ne* estimates suggesting that additional mechanisms influence the range of *Ne*.

### Maximal growth rate correlates negatively with *Ne*

**Fig. 3:**
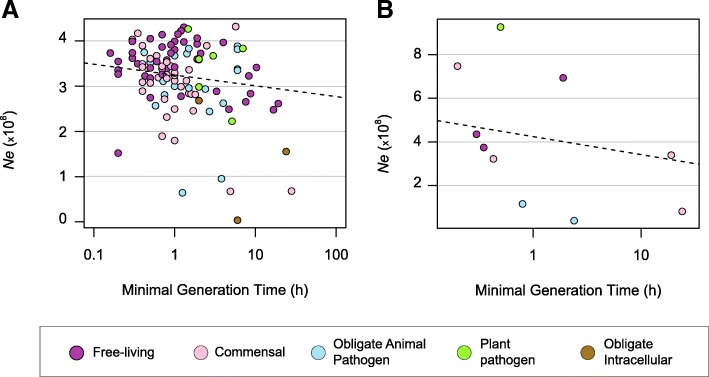
Correlation between bacterial growth rate and effective population size. **a**. Correlation between estimates of *Ne* based on *dS/dN* **(**as presented in Fig. 1**)** and growth rates (Spearman’s rho = − 0.38, *P* < 10^− 4^, PIC correction). **b**. Correlation between absolute estimates of *Ne* and growth rates (Spearman’s rho = − 0.59, *P* = 0.08). Growth rates were defined as reported minimal doubling times (Additional file 19: Table S5)

Although the reported doubling times might not accurately reflect the true growth rate of bacteria under natural conditions, we observed a negative correlation between the minimal doubling time of bacteria and *Ne* (Fig. 3a Spearman’s rho = − 0.38, *P* < 10^− 4^, PIC correction). Note that when doubling time correlates negatively with *Ne*, its inverse (i.e., growth rate) must correlate positively with *Ne*. Similar results were observed when *Ne* was estimated on core genomes (Additional file 5: Figure S4, Spearman’s rho = − 0.22, *P* < 0.05, PIC correction). The same, but not significant, trend was found for the few species for which we could estimate absolute estimates of *Ne* (Fig. 3b Spearman’s rho = − 0.59, *P* = 0.08). These results indicate that those species capable of rapid growth typically have larger effective population sizes.

### Recombination has limited impact on *Ne*

Asexual organisms should display reduced effective population sizes due to genomic linkage [9, 13], since strongly linked genomes are expected to lose neutral polymorphisms through background selection, hitchhiking and/or Müller’s ratchet [8]. However, bacteria engage in homologous recombination to varying degrees—ranging from strictly clonal species to highly recombining taxa [10, 14]—and the extent to which recombination effectively modulates the levels of bacterial polymorphisms is unknown.

We tested how the scale of recombination, estimated both by ClonalFrameML (*r/m*) [15] and by the ratio of homoplasic to non-homoplasic alleles (*h/m*), [10] affects estimates of *Ne*. Both methods for detecting recombination were highly correlated to one another (Additional file 6: Figure S5A-B); however, *h/m* ratios are much more consistent between the core genes and the set of universal genes from the same species (Additional file 6: Figure S5-D). With either metric, there is little if any association between recombination rate and *Ne* (Additional file 7: Figure S6).

### *Ne* drives the evolution of the pan-genome

**Fig. 4:**
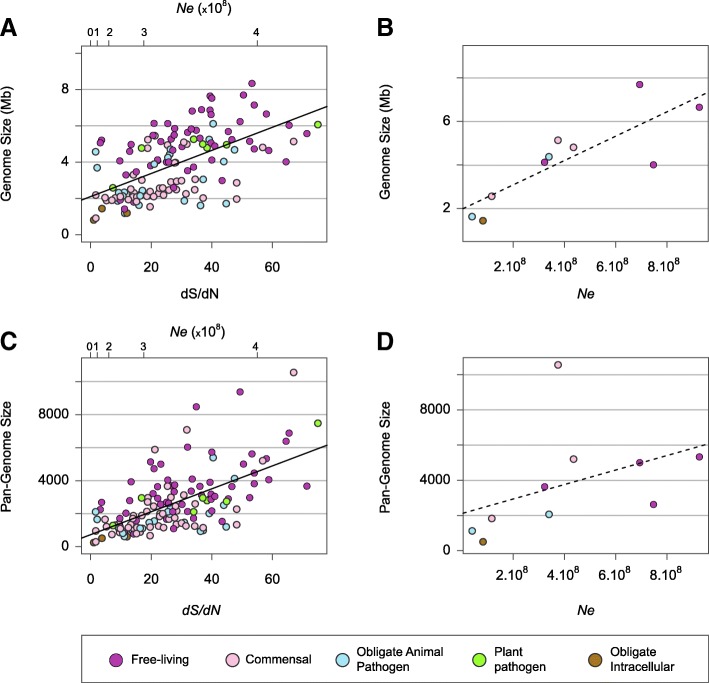
Correlation between effective population size, genome size and pan-genome size. **a**. Genome size vs. *Ne*. Genome size for a given species was calculated as the average across all sequenced strains, and *Ne* was computed from the average *dS/dN* ratio determined for common set of universally distributed genes (as described in Fig. 1 and Methods, Spearman’s rho = 0.30, *P* < 0.001, PIC correction). Colors indicate the life style of each species: green: free-living, blue: commensal; red: obligate animal pathogen, light green: plant pathogen, purple: obligate intracellular. **b** Genome size vs. absolute estimates of *Ne* (Spearman’s Rho = 0.77, *P* < 0.05). Genome size for a given species was calculated as the average across all sequenced strains, and *Ne* was computed for the species with known mutation rates using Watterson’s estimator. **c** Pan-genome size vs. *Ne.* Pan-genome size for a given species was calculated as the total number of protein families detected normalized by strain number, and *Ne* was computed from the average *dS/dN* ratio determined for common set of universally distributed genes (Spearman’s rho = 0.48, *P* < 10^− 8^, PIC correction). **d** Pan-genome size vs. absolute estimates of *Ne*

Previous analyses reported a strong negative association between the level of drift and bacterial genome size [3], and we observe much the same trend (Fig. 4a): bacterial species with larger *Ne* (less subject to drift) have larger genome sizes (Spearman’s rho = 0.30, *P* < 0.001, PIC correction). The same result was obtained when *dS/dN* ratios are calculated from the core genomes (Additional file 8: Figure S7A, Spearman’s rho = 0.32, *P* < 0.001, PIC correction) or based on the absolute estimates of *Ne* (Fig. 4b, Spearman’s Rho = 0.77, *P* < 0.05). This positive relationship between *Ne* and genome size persists when confining analyses to bacteria in each of the lifestyle categories (free-living, commensals and obligate pathogens) (Additional file 9: Figure S8); however, the correlations no longer reach significance after PIC correction. These results are in line with previous studies [3], supporting the view that the higher effectiveness of selection in bacteria with large population sizes is linked to larger genome sizes.

Prokaryote genomes are largely devoid of intergenic and nonfunctional DNA, such that larger genomes are usually enriched in functional accessory genes [16]. Pan-genome size (i.e., the total number of genes encoded by a species adjusted for strain number to allow comparisons across species) correlates positively with *Ne*, as estimated both from universally distributed genes (Fig. 4c, Spearman’s rho = 0.48, *P* < 10^− 8^, PIC correction) and from core genomes (Additional file 8: Figure S7B, Spearman’s rho = 0.48, *P* < 10^− 7^, PIC correction), and these correlations remain significant after PIC correction for each major lifestyle category (Additional file 10: Figure S9, Additional file 11: Table S2). This correlation remained significant when the size of the pan-genome was corrected for the number of strains by an alternate method (Additional file 12: Figure S10, Spearman’s rho = 0.48, *P* < 10^− 8^, PIC correction). Pan-genome sizes and average genome sizes are strongly correlated (Additional file 13: Figure S11) making it difficult to disentangle whether drift impacts the size of individual bacterial genomes or drives the gene diversity of bacterial species. However, the strength of the correlations between *Ne* and pan-genome size are systematically better than those between *Ne* and average genome size (Additional file 11: Table S2), suggesting that pan-genome size—rather than individual genome size—is being shaped by the efficacy of selection.

Because we evaluated numerous features of bacterial genomes and lifestyles, we performed several multivariate analyses to characterize the interactions among all the quantitative variables examined in this study. The first two principal axes obtained in a principal component analysis (PCA) of the variables represented 64% of the variance, with PC1 associated with genome size, pan-genome size, *Ne,* and GC-content (Additional file 14: Table S3) and PC2 associated with the maximal growth rate and the recombination rate (*h/m*). Similar results were obtained when *Ne* was estimated on the core genome (Additional file 14: Table S3) and when *Ne* was estimated without restraining the set of strains based on *dS* values (Additional file 14: Table S3). We then built the matrix and corresponding network of correlations across these quantitative variables (Additional file 14: Table S3, Additional file 15: Figure S12), and again, *Ne*, pan-genome size and genome size were strongly correlated (Additional file 14: Table S3, Additional file 15: Figure S12). Furthermore, GC-content was strongly correlated with genome size, weakly correlated with pan-genome size and showed no association with *Ne*. In sum, these analyses indicate that our different estimates of *Ne* are systematically and most strongly associated with pan-genome size.

### Fine-scale dynamics of genome evolution

**Fig. 5:**
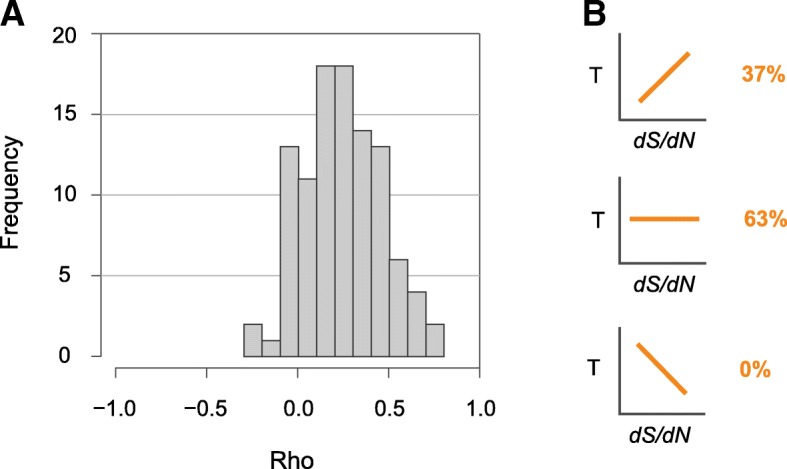
Correlations between gene turnover and effective population size. **a**. Gene turnover, *T*, was defined as the rate of gene gains divided by the rate of gene losses at each branch of each species tree. Rates of gene gains and losses were estimated using a posterior probability threshold of 0.2. For each branch of the same species trees, the *dS/dN* ratio was estimated using CodeML (see Methods), and the Spearman’s correlation between *T* and *dS/dN* ratios computed for each species. The distribution of the coefficient *rho* across species is represented. **b.** Species were organized into three categories: those with a positive correlation between gene turnover *T* and *dS/dN* (top, Spearman’s correlation, *P* < 0.05); those with no significant correlation between *T* and *dS/dN* (middle; Spearman’s correlation, *P* ≥ 0.05); and those with a negative correlation between *T* and *dS/dN* (bottom, Spearman’s correlation, *P* < 0.05)

As reported in previous studies, [3, 17], we show that bacterial genes are typically lost by drift when selection is relaxed. Since the evolution of bacterial genomes frequently involves the loss and gain of multiple genes, we hypothesized that events of gene loss would correspond to episodes of more relaxed selection. To test this assumption, we built a phylogenetic tree for each species based on its core genome, and then estimated rates of gene gains and gene losses along each branch using Count [18]. For each branch, we calculated a rate of gene turnover *T* (defined as the ratio of the rate of gene gains divided by the rate of gene losses) and a *dS/dN* ratio (see Methods). We observed a positive correlation between *dS/dN* and the rate of gene turnover for the vast majority of species (Fig. 5a). This correlation reached significance in 37% of species and in no cases did we observe a significant negative correlation (Fig. 5b). Similar results were obtained when inferring gene losses and gene gains under different parameters (Additional file 16: Figure S13). Species evolving under less efficient selection (i.e., those with lower *dS/dN* values) were those in which gene losses outnumbered gene gains (*T* ≈ 0), whereas gene content was more stable or increased (*T* > > 0) in species evolving under more effective selection (i.e., those with higher *dS/dN* values). Together, these results suggest that species subjected to stronger drift display a net loss of genes and are unlikely to maintain a large pan-genome.

## Discussion

The size and organization of bacterial genomes are governed by population-level processes dictating a need for accurate estimates of effective population size (*Ne*). However, estimating the effective population sizes of microbial species has been notoriously difficult on account of several factors—the enormous sizes of their census populations, the variation in the amount of recombination among lineages, and the constraints on what constitutes a species.

Due to their size, asexuality and short generation times, bacteria have tremendous potential for population growth and can attain extremely large population numbers even in very circumscribed environments. But because such populations are typically founded by one or few individuals, there are likely to be substantial differences between the standing and the effective population sizes in most bacterial species [9]. This disparity was initially noted by those assessing the variation within natural populations of *E. coli* [19, 20], and it is particularly evident when considering marine bacteria, which are the most abundant cellular organisms on the planet [5, 21]. Based on *dN/dS* ratios, we estimated effective population sizes on the order of 10^8^ for *Prochlorococcus marinus,* whereas its census populations may be upwards of 10^13^ [5]. This disparity was also noted by Batut et al. (2015) [22], and several explanations, including population substructure, frequent selective sweeps and background selection, have all been invoked to account for these discrepancies [9, 10, 20, 23]. We note that our analyses were restricted to the genomes classified as *Prochlorococcus marinus*, whereas other studies have included all genomes typed to *Prochlorococcus* when analyzing this “species” [5]. Defining species of *Prochlorococcus* is particularly problematic and inconsistent across studies, since this group represents a single species based on 16S rRNA sequence (i.e., > 97% identity) but comprises multiple species based on Average Nucleotide identity (ANI), which can be as low as 66% [24]. Although the classification of genomes into species should not be based solely on 16S rRNA sequences, many genomic studies ignore such guidelines or do not attempt to characterize the taxonomic level of the studied populations, which hampers comparisons across studies. Aside from the natural forces that might contribute to the relatively small *Ne* of bacteria, it is also possible that *Ne* estimates based on neutral variation are inaccurate because synonymous sites are possibly under effectively stronger selective constraints in very large populations [5].

In asexual microbes, genomic linkage can potentially cause the loss of neutral variants through Hill-Robertson effects, thereby reducing estimates of *Ne* [9, 13]. Because recombination varies widely among microbial species [14], sometimes approaching the levels of outcrossing, sexual species [25–27], we predicted that Hill-Robertson effects would be most evident in purely clonal species, which, in turn, would have the smallest *Ne*. However, we detected no significant effect of recombination rate on *Ne* despite the theoretical predictions made for bacteria, and empirical results observed in animals and plants [9, 13, 28–30]. The absence of a relationship between recombination and *Ne* in prokaryotes could result from relatively rapid changes in *Ne* or rates of recombination over the evolutionary history of a species, which would prevent us from capturing long-term estimates of *Ne* and/or recombination rates by analyzing the variation in contemporary populations.

In animals, *Ne* increases with progeny size but is poorly predicted by geographic range [4]. Similarly, growth rate in bacteria, which is somewhat comparable to progeny size of animals, can drive the evolution of *Ne*, since fast growing bacteria can readily reach larger population sizes. It also appears that the ecological niche occupied by a given bacterial species can impose constraints on their growth ability. Thus, the growth rate and lifestyle of natural populations seem to be the primary forces shaping *Ne* in microbial species.

The relationship between *Ne* and genome size in bacteria is well established: bacterial species with the smallest effective population sizes are those with the smallest and most compact genomes. For example, the small genomes of pathogens and symbionts have usually been considered to result from drift, imposed through the repeated bottlenecks occurring during infection of new hosts, which reduces the efficacy of selection [20]. This causes the inactivation of many previously useful genes, which erode and are eliminated by the overriding mutational bias towards deletions, resulting in a small and compact genome [3, 31].

Why does *Ne* drive the diversity of gene repertoires in prokaryotic species? The most intuitive explanation stems from the drift-barrier model, which was originally proposed to explain how increased levels of drift render selection ineffective to modulate rates of mutations [32]. The pan-genome of bacterial species consists mainly of “accessory” genes, those harbored by relatively few strains and not part of the essential core genome [16, 33]. Our model predicts that lower *Ne* (i.e., higher levels of drift) will increase the stochastic loss of accessory genes, especially those of little benefit to fitness. In this scenario, all accessory genes are expected to be beneficial under some conditions, and based on nearly-neutral theory, these genes will be maintained only when their selection coefficients can overcome the intensity of drift (*i.e*, *s* > > 1/*Ne)* [34]. As such, species with small *Ne* retain the most beneficial accessory genes, whereas larger numbers of accessory genes of more modest fitness contributions will be conserved by selection in species with larger *Ne*. This model is particularly relevant in prokaryotes, since accessory genes can be exchanged across species boundaries by horizontal gene transfer, thereby increasing the scale and speed with which gene repertoires can increase.

Our model can potentially explain the discrepancy observed in marine bacteria that have small genomes (usually under 2 Mb) but are considered to have extremely large population sizes [21]. Interestingly, the pan-genomes of such taxa (e.g., *Prochlorococcus*) are thought to be enormous, despite the small size of individual genomes [5], which suggests that they adhere to our drift-barrier model of pan-genome evolution. To date, relatively few genomes are available for these taxa and, more importantly, the taxonomy of these organisms is highly debated, making it difficult to assign species boundaries [24]. A more extensive analysis of these taxa and their species borders would help elucidate this issue.

A key aspect of our model is the assumption that the vast majority of genes in bacterial genomes are adaptive. Multiple lines of evidence suggest that bacteria tend to lose deleterious and neutral sequences very rapidly, as evident from the very small amount of intergenic DNA, pseudogenes, introns and mobile elements in prokaryotes [35], and recent modelling further supports the view that most accessory genes are beneficial [31]. Although mobile elements, such as temperate phages, can represent a substantial fraction of bacterial pan-genomes, these elements often carry beneficial functions to their bacterial host and are conserved by purifying selection [36]. That the vast majority of genes constituting the pan-genome are beneficial does not mean that each will be conserved by selection because genes with small selection coefficients can be lost through drift. As a consequence, the size of pan-genome is expected to be a function of drift, and, therefore, of *Ne*.

Two recent publications have attempted to evaluate the interplay between effective population size and pan-genome size. The first detected a positive correlation between genome polymorphism diversity and pan-genome size [17], results that are in general agreement with our conclusions; but this study derived estimates of *Ne* from neutral polymorphisms, under the untested assumptions that species’ borders are well defined and that mutation rates did not deviate among species, which limits the robustness of their findings. In contrast, the second study hypothesized that pan-genome size resulted from the combined effects of effective population size and the potential for migration to new niches [37]. Although we show that pan-genome size is largely consistent with a drift-barrier model, the authors dismiss this alternative by presupposing that pan-genomic sequences must be neutral in order to be shaped by drift and that neutral sequences would be purged from the pan-genome by the mutational bias towards deletions. A more accurate interpretation of the impact of drift on pan-genome size is that effective population size modulates the efficacy of selection, thereby affecting the number of genes that are effectively perceived as neutral (and eliminated) and the number of genes that are retained by selection (the pan-genome).

Our model of pan-genome evolution contends that the vast majority of genes in a genome are maintained by selection, because neutral and non-adaptive regions are removed by the deletion bias inherent to bacterial genomes [38, 39]. Recently, others have reasoned that a neutral (or nearly-neutral) model of pan-genome evolution is more parsimonious, since populations with higher *Ne* are expected to sustain higher numbers of nearly-neutral alleles (i.e., nearly-neutral variants in gene content) [36, 40, 41]. Along these lines, Vos et al. [41] considered it unlikely that the entire pan-genome is adaptive since mobile elements often constitute a large part of the pan-genome [42]. However, close inspection of mobile elements reveals that many encode prophages [16], which mostly encode proteins involved in their own replication and morphogenesis, but could help bacteria eliminate competitors [43], and are maintained by selection [36]. Other types of mobile elements can expand briefly after reduction in *Ne* but most of them are eventually eliminated by deletions [44]. Additionally, if pan-genomes were guided by neutral evolution, those species with higher *Ne* should also harbor large amounts of other types of nearly-neutral sequences, such as intergenic DNA and pseudogenes, which is not the case. In contrast, we do not observe an increase in the amount of intergenic DNA with *Ne* (Additional file 17: Figure S14), as estimated from *dS/dN* values and pseudogenes are equitably rare in bacterial species on account of their removal by deletions [45, 46].

Prokaryotic species possess genomes and pan-genomes in which virtually all genes are maintained by selection, such that species with larger effective population sizes sustain larger pan-genomes. Additionally, the non-functional regions within prokaryotic genomes are transient denizens that are eventually purged by deletions. As such, strains within a prokaryotic species can differ substantially in their gene contents due to differential gene acquisition and loss. In contrast, eukaryotic species display the opposite trend in which less effective selection (i.e., lower *Ne*) is associated with larger genomes, which expand through the accumulation of non-coding and slightly deleterious DNA, such as introns, mobile elements and intergenic DNA [2]. As a result, differences in genome size among eukaryotes need not be associated with changes in gene contents, and the gene repertoire in all members of species are identical or very nearly so. The processes underlying the disparate trends of prokaryotes and eukaryotes are three-fold: first and foremost is the pervasive mutational bias in prokaryotes towards deletions, which rids genomes of non-functional DNA even in species where selection is abated; second is the limited ability of eukaryotes to gain genes through horizontal gene transfer [47–49], which offers prokaryotes rapid opportunities for changes in gene contents and functional capabilities; and third is sexual reproduction, in which conserved blocks of genes and chromosome numbers are required for homologous exchange, thereby serving to homogenize genome contents within eukaryotic species.

## Conclusions

In this study, we provide estimates of *Ne* for a large set of prokaryotic species, and show that *Ne* is shaped by lifestyle and growth rate, but is not substantially impacted by phylogenetic relationships or recombination rates. We further show that the size of bacterial pan-genomes, i.e.*,* the total number of genes harbored by a species, rather than the size of individual genomes, is driven by *Ne*. Whereas recent publications have debated whether the size of the pan-genome is driven by adaptive or neutral processes, we propose that pan-genome size is guided by drift-barrier evolution. This model emphasizes that accessory genes of little adaptive value (i.e.*,* genes with low selection coefficients *s*) are virtually neutral when drift dominates over selection and that such genes are eventually lost due to the pervasive deletion bias occurring in prokaryotic genomes. Since *Ne* determines the amount of genes that are perceived as effectively neutral, species with large *Ne* are able to retain larger gene pools than species with low *Ne*.

## Methods

### Species sampling and strain classification

Based first on the species assignments and designations at the NCBI website (ftp.ncbi.nlm.nih.gov/genomes/; April 2016), we downloaded all bacterial and archaeal species (*n* = 245) represented by at least 15 genome sequences. For each genome, we used HMMER v3.1b2 [50] to recover the set of 44 proteins reported as being universally distributed in prokaryotes [51]. The best hit of each protein in each strain (*e*-value < 10^− 5^) was considered a potential ortholog. Because several genomes contain paralogs that might lead to the misidentification of orthologs (such that the true ortholog is missing from the genome but a paralog is present), for each of the universally distributed homologs, we assembled for each species the distribution of *e*-values of confirmed paralogs (the second-or-higher best hit in each strain with an *e*-value < 10^− 5^) and the distribution of *e*-values for potential orthologs (the best hit in each strain). A potential ortholog was considered a true ortholog when its *e*-value_log_ was more similar to the median *e*-value_log_ of potential orthologs than to the mean *e*-value_log_ of paralogs. Strains in which we detected less than 42 universally distributed orthologs were excluded since they likely represent incomplete assemblies. (In the case of *Mycoplasma*, four missing proteins were tolerated since it harbors a highly reduced genome.)

Each protein family was aligned with MAFFT v7 [52] and transformed in silico into the corresponding nucleotide sequences, and alignments were merged into a single concatenate for each species. For each concatenate, we computed the pairwise distances *D* using RAxML v8 under a GTR + Γ model [53]. Many species contained multiple strains that were identical (or nearly so), so we randomly excluded strains with very short evolutionary distances (*D* < 0.00005). After these procedures, all named species represented by < 15 strains were excluded from the analysis, and final dataset comprised 152 bacterial species and one archaeon.

Several individual species contained very large numbers of sequenced strains (e.g., *Escherichia coli, Mycobacterium tuberculosis, Pseudomonas aeruginosa, Salmonella enterica, Staphylococcus aureus and Streptococcus pneumoniae*). Those species with > 400 distinct strains were randomly subsampled down to 400 strains. We then defined species borders based on gene flow, as described in a previous methodology [10]. A total of 44 species (29%) were redefined, such that sexually isolated strains were removed from the species. The list of included and excluded strains for each species is detailed in Additional file 18: Table S4.

### Phylogeny

We built a phylogenetic tree including the entire set of species based on the sequences of the universally distributed proteins. For each species, we randomly selected one strain from among those with the most complete representation of the 44 proteins. Each set of universally distributed orthologous proteins were aligned with MAFFT v7 [52], trimmed using BMGE [54] with the BLOSUM30 matrix and merged into a single concatenate. The phylogenetic tree was built using a maximum likelihood approach in RAxML v8 [53] under the LG + Γ model. We computed 100 rapid bootstrap replicates using the same model [55]. The resulting tree was used to correct statistical tests with the phylogenetic independent contrast method (PIC) [56] implemented in the R package Ape [57].

### Defining pan- and core genomes

For each genetically defined species, we initially identified orthologous proteins for each pair of strains using Usearch Global v8.0 s [58]. Orthologs were defined as best reciprocal hits with at least 70% protein sequence identity and 80% length conservation. Orthologous proteins were then grouped into protein families by transitivity, such that every pair of orthologs belongs to the same protein family. The total number of protein families—including families consisting of a single, unique protein—defines the size of the pan-genome (*N*_*pan*_) of each species. Because the size of the pan-genome increases with the number of sampled strains [16], it cannot be compared directly among species with different sample sizes. To compare pan-genome sizes across species, we defined *P*, the normalized pan-genome size, corrected as in [59] such that *P = N*_*pan*_*/α*, where *α* is the sum of harmonic series of the number of strains defined by $\alpha =\sum \limits_{\mathrm{i}=1}^{\mathrm{n}-1}\frac{1}{i}$, with *n* representing the number of strains for a given species. The pan-genome was estimated with a second metric, *Ps*, in which each species was subsampled to the same number of strains (*n* = 13) while maximizing strain divergence calculated on the core genome (i.e., the pan-genome of the13 most divergent strains of each species). Protein families were considered as part of the core genome if present in ≥85% of the strains in the species, and those protein families with paralogs were systematically excluded from the core genome. Each core protein family was aligned with MAFFT v7 [52] and reverse-translated in silico into the corresponding nucleotide sequences. The alignments were merged into a single concatenate of core genes for each species.

### Absolute estimates of *Ne*

We computed Watterson’s estimator *θ* [59] with Pegas v0.9 [60] on four-fold degenerate sites within each concatenate of the core genome. Effective population size (*Ne*) was given by *θ = 2.N*_*e*_*.μ* [59], where *μ* represents the mutation rate, as available for 10 bacterial species [11, 12].

### Estimation of *Ne* based on *dN/dS*

For each species, pairwise *dN/dS* ratios were computed with PAML v4.3b using the *yn00* algorithm [61, 62] on the concatenate of universally distributed genes or the concatenate of core genes. Since *dN/dS* ratios do not remain constant over time [7], this metric does not allow for direct comparisons of *Ne* across species unless the strains within a species diverged within the same time interval. Therefore, for each species, we computed the average *dN/dS* for the pairs of strains having *dS* values in the range of 0.1 ≤ *dS* ≤ 0.3, which was well below saturation and maximized the number of analyzed species. Absolute estimates of *Ne* were inferred from *dN/dS* ratios following [63]: $\frac{dN}{dS}=\frac{N_{e^S}}{1={e}^{-{N}_{e^S}}}$. In this case, the selection coefficient *s* is likely to be similar across species since it applies to the same set of universally distributed genes. We used the 10 absolute values of *Ne* defined above to estimate the selection coefficient *s* of the universal set of genes. *Ne* estimates for each species were then determined using the mean value of the different estimates of the selection coefficient *s*.

### Estimating recombination rates

We used two approaches to estimate recombination rates across the core genomes and the concatenates of universally distributed genes of all species. First, we built the phylogenetic tree of each species using RAxML v8 under a GTR + Γ model on the two datasets and we estimated the transition/transversion ratio *kappa* with the same program. We then used ClonalFrameML [15] to estimate *r/m* across both datasets, where *r* is the number of alleles introduced or exchanged by recombination and *m* is the number of alleles introduced by mutations. We also used the ratio *h/m* to estimate recombination rates [10], where *h* is the number of homoplasic alleles and *m* the number of non-homoplasic alleles in the two datasets.

### Growth rates

Minimal doubling times were obtained from the literature (Additional file 19: Table S5). Several of these values had already been assembled in [64]. In cases where there were multiple growth-rate estimates for a species, we used the smallest doubling time reported.

### Gene turnover

Rates of gene loss and gene gain were estimated per branch along the tree built for each species using Count [18]. Based on the pan-genome (defined above), we generated a matrix of gene presence or absence across all strains within each species. Rates of gene gains and losses were estimated based on a Poisson distribution. One hundred rounds of rate optimization were computed. Ancestral reconstructions were performed using posterior probabilities, with rates of gains and losses initially estimated by maximum likelihood with posterior probability thresholds of 0.2 and 0.3. Results presented use a posterior probability threshold of 0.2, since it was shown to be the most accurate when run on similar data sets [65]. For each branch *i*, the gene turnover *T*_*i*_ was defined as *T*_*i*_ *= G*_*i*_*/L*_*i*_ with *G*_*i*_ denoting the branch rate of gene gains and *L*_*i*_, the rate of gene losses. The same set of species trees was then used to estimate *dN/dS* ratios along the branches of the trees using CodeML implemented in PAML v4.3b [62] with the free-ratios model. Due to the extensive computation time required, the core genome concatenate of each species was subdivided into 150,000 bp fragments, which were each used to infer *dN/dS* ratios. Initial *dN/dS* ratios were set with the *dN/dS* ratio estimated for each species under the *yn00* algorithm (see above). For each branch *i* of each species tree, *dN/dS* ratios were defined as the average *dN/dS* of the branch *i* estimated across the different fragments of the core genome. Due to the size of the dataset, not all fragments and species (i.e., those containing over 60 strains) could be evaluated with CodeML, with the result that a total of 102 species were analyzed.

## Additional files

Additional file 1: **Table S1**. Data summary. (XLSX 84 kb)

Additional file 2: **Figure S1.** Correspondence between *Ne* estimated from universally distributed genes and from the complete set of core genes. Effective population sizes are estimated from *dS/dN* considering a common set of universally distributed genes for each species (*x*-axis) and the entire set of core gene set for a species (*y*-axis). The dashed line represents the theoretical expectation (*y = x*). Most species present similar estimates of *Ne* when computed on both sets of genes with the exception of *Aggregatibacter actinomycetemcomitans*, *Vibrio alginolyticus* and *Vibrio cyclitrophicus*. (PDF 141 kb)

Additional file 3: **Figure S2.** Correlation between phylogenetic distance and *Ne* dissimilarity. Phylogenetic distances for each pair of species were obtained from the maximum likelihood species tree (Fig. 1). Dissimilarity in effective population sizes for each species pair is defined as *|Ne*_*i*_ *- Ne*_*j*_*|* for species *i* and *j*, respectively. (PDF 8178 kb)

Additional file 4: **Figure S3.** Association between bacterial lifestyle and effective population size, as computed from species’ core genomes. Lifestyle colors and designations follow those presented in Fig. 1, with the number of species in each lifestyle category indicated. ***P < 0.001, **P < 0.01, *P < 0.05, Wilcoxon test. (PDF 119 kb)

Additional file 5: **Figure S4.** Correlation between growth rate and effective population size computed from species’ core genomes. Growth rates are defined as minimal doubling times reported in the literature (Additional file 19: Table S5). Spearman’s rho = − 0.22, *P* < 0.05, PIC correction. (PDF 135 kb)

Additional file 6: **Figure S5.** Comparison of recombination detection methods. Recombination rates were estimated based on the ratio of homoplasic to non-homoplasic alleles (*h/m*) [10] and with ClonalFrameML (*r/m*) [15]. The two methods were compared on the set of universal genes (A) or on the entire core genome (B) for each species. The performance of each method was then evaluated by comparing the recombination rate on the set of universal genes relative to the complete core genome of each species with *h/m* ratios (C) and *r/m* ratios (D). Spearman’s correlation coefficients rho are indicated on top of each graph. (PDF 382 kb)

Additional file 7: **Figure S6.** Impact of recombination on estimates of effective population size. Relationship between recombination rate and the effective population size of each species. Recombination rate, estimated from the frequencies of homoplasies, and *dS/dN* for each species were calculated for universally distributed genes (A) and for the set of core genomes (C), and recombination rate, estimated with ClonalFrameML, and *dS/dN* for each species were calculated for universally distributed genes (B) and fore the set of core genomes (D). (PDF 359 kb)

Additional file 8: **Figure S7.** Correlation between genome size, pan-genome size, and effective population sizes as computed from core genomes. Correlation between genome sizes (A) and pan-genomes sizes (Spearman’s rho = 0.32, *P* < 0.001, PIC correction) (B) when *N* average *dS/dN* ratios are determined for core set of genes for each species (Spearman’s rho = 0.48, *P* < 10^− 7^, PIC correction). (PDF 240 kb)

Additional file 9: **Figure S8.** Correlation between genome size and *Ne* for each lifestyle category. Genome size for a given species was calculated as the average across all sequenced strains. *dS/dN* ratios were calculated from the common set of universally distributed gene (A–C) and from the core genome of each species (D–F). Spearman’s correlations were adjusted with phylgenetically independent contrasts. (PDF 278 kb)

Additional file 10: **Figure S9.** Association between pan-genome size and *Ne* for each lifestyle category. Pan-genome size for a given species was calculated as the total number of protein families detected normalized by strain number. *dS/dN* ratios were calculated from the common set of universally distributed gene (A–C) and from the core genome of each species (D–F). Spearman’s correlations were adjusted with phylogenetically independent contrasts. (PDF 274 kb)

Additional file 11: **Table S2**. Correlation statistics between *Ne* and genome or pan-genome size for each lifestyle category. (XLSX 45 kb)

Additional file 12: **Figure S10.** Association between *Ne* and pan-genome size, adjusted for sample size (Spearman’s rho = 0.48, *P* < 10^− 8^, PIC correction). *Ne* was estimated from *dS/dN* ratios (Fig. 1). Pan-genome sizes were corrected for sample size by analyzing the same number of genomes for each species while maximizing the divergence rate of the core genome. Using a recursive approach, the pair of the most similar genomes for a species was identified, and one of the two genomes was randomly discarded. This process was repeated until each species was down-sampled to 13 genomes. The pan-genome was then re-built for each species as described above. (PDF 154 kb)

Additional file 13: **Figure S11.** Association between genome size and pan-genome size. Genome sizes represent averages a across all sequenced strains for a given species and pan-genome sizes were calculated as the total number of protein families normalized by the number of strains of a given each species. (PDF 138 kb)

Additional file 14: **Table S3**. Principal component analysis statistics of the quantitative variables used in the study. (XLSX 13 kb)

Additional file 15: **Figure S12.** Network of correlations among genomic and lifestyle variables. The correlation network was built using the *P*-values obtained from the correlation matrix for these quantitative variables (Additional file 14: Table S3). Each node represents a quantitative variable, and the thickness of edges is proportional to the strength of the correlations, defined as –log(*P*-value). Correlations with *P*-values > 0.01 were not included in the network. (PDF 112 kb)

Additional file 16: **Figure S13.** Correlation between gene turnover and effective population size. A. Gene turnover, *T*, was defined as the rate of gene gains divided by the rate of gene losses on each branch of each species tree. Rates of gene gains and losses were estimated with a posterior probability threshold of 0.3. For each branch of a species trees, the *dS/dN* ratio was estimated using CodeML (see Methods). The Spearman’s correlation between *T* and *dS/dN* ratios was computed for each species, and he distribution of the coefficient *rho* across species is presented. B. Species were organized into three categories: those with a positive correlation between gene turnover *T* and *dS/dN* (top, Spearman’s correlation, *P* < 0.05); those with no significant correlation between *T* and *dS/dN* (middle; Spearman’s correlation, *P* ≥ 0.05); and those with a negative correlation between *T* and *dS/dN* (bottom, Spearman’s correlation, *P* < 0.05). (PDF 119 kb)

Additional file 17: **Figure S14.** Correlation between *dS/dN* and intergenic DNA. For each species, *dS/dN* ratios were estimated as in Fig. 1. Average intergenic DNA of each species corresponds to the average number of base pairs between two consecutive protein-coding genes. No positive correlation was observed between *dS/dN* and the average intergenic DNA (Spearman’s rho = − 0.22, *P* < 0.05). (PDF 137 kb)

Additional file 18: **Table S4**. List of analyzed genomes for each species. (XLS 317 kb)

Additional file 19: **Table S5**. Minimum doubling times of each species and corresponding references. (DOCX 217 kb)
