# Supplementary figures and images for: Factors driving effective population size and pan-genome evolution in bacteria
Source: BMC Evol Biol. 2018 Oct 12;18:153. doi: 10.1186/s12862-018-1272-4 (PMC6186134; doi:10.1186/s12862-018-1272-4)

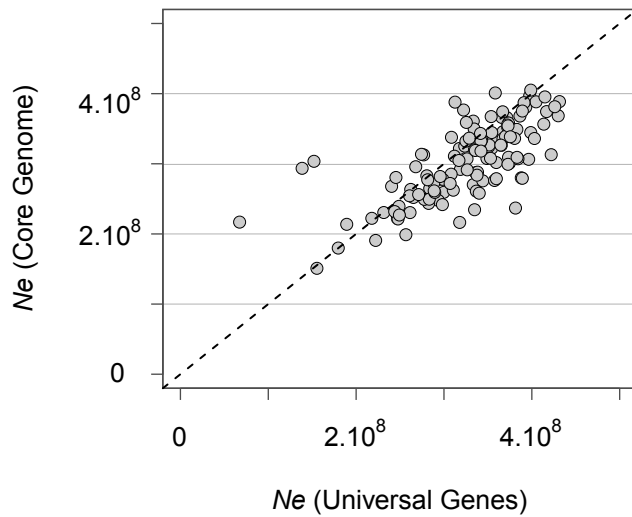

Supplement: Supplementary file 2 — Figure S1. Correspondence between Ne estimated from universally distributed genes and from the complete set of core genes. Effective population sizes are estimated from dS/dN considering a common set of universally distributed genes for each species (x-axis) and the entire set of core gene set for a species (y-axis). The dashed line represents the theoretical expectation (y = x). Most species present similar estimates of Ne when computed on both sets of genes with the exception of Aggregatibacter actinomycetemcomitans, Vibrio alginolyticus and Vibrio cyclitrophicus. (PDF 141 kb) [file 12862_2018_1272_MOESM2_ESM.pdf]

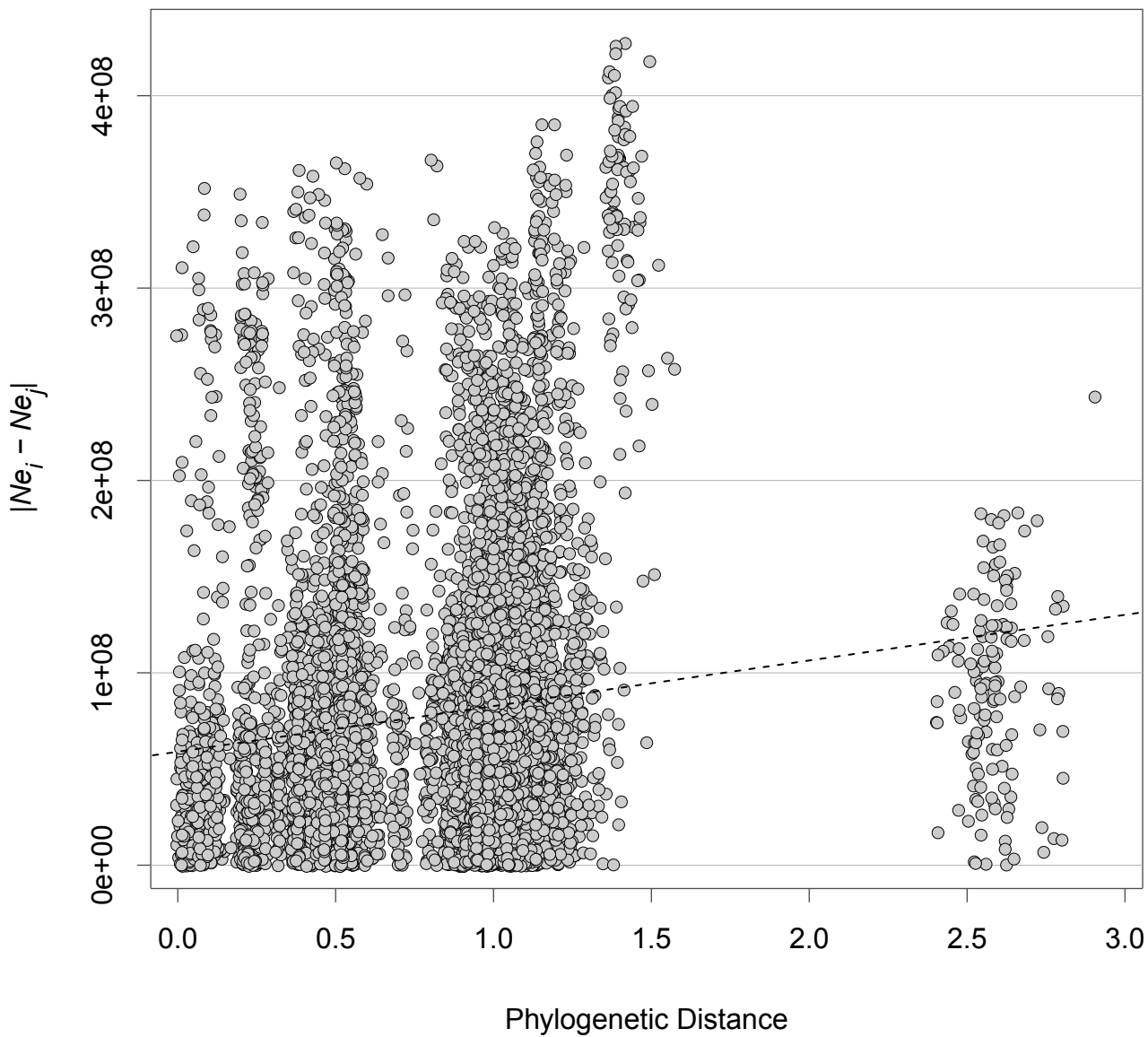

Supplement: Supplementary file 3 — Figure S2. Correlation between phylogenetic distance and Ne dissimilarity. Phylogenetic distances for each pair of species were obtained from the maximum likelihood species tree (Fig. 1). Dissimilarity in effective population sizes for each species pair is defined as |Nei - Nej| for species i and j, respectively. (PDF 8178 kb) [file 12862_2018_1272_MOESM3_ESM.pdf]

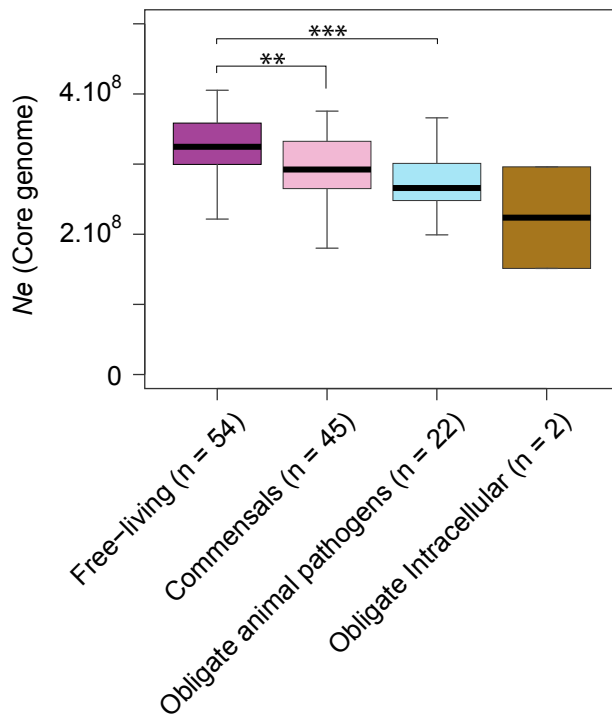

Supplement: Supplementary file 4 — Figure S3. Association between bacterial lifestyle and effective population size, as computed from species’ core genomes. Lifestyle colors and designations follow those presented in Fig. 1, with the number of species in each lifestyle category indicated. ***P < 0.001, **P < 0.01, *P < 0.05, Wilcoxon test. (PDF 119 kb) [file 12862_2018_1272_MOESM4_ESM.pdf]

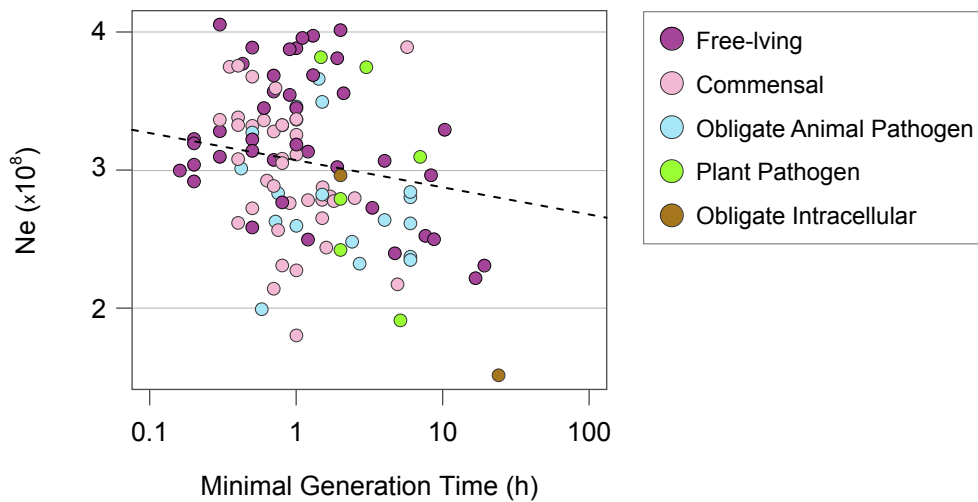

Supplement: Supplementary file 5 — Figure S4. Correlation between growth rate and effective population size computed from species’ core genomes. Growth rates are defined as minimal doubling times reported in the literature (Additional file 19: Table S5). Spearman’s rho = − 0.22, P < 0.05, PIC correction. (PDF 135 kb) [file 12862_2018_1272_MOESM5_ESM.pdf]

A

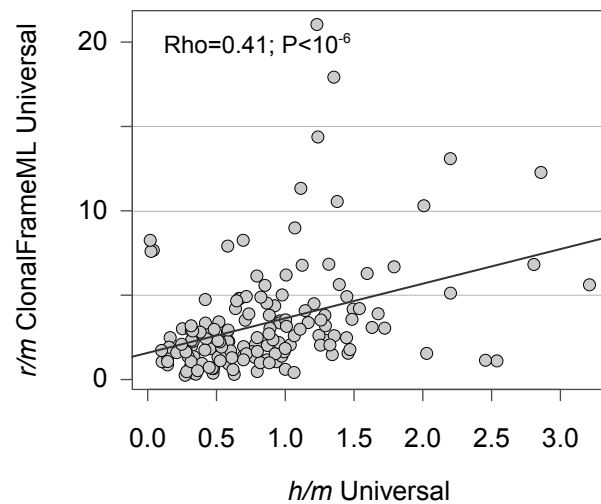

B

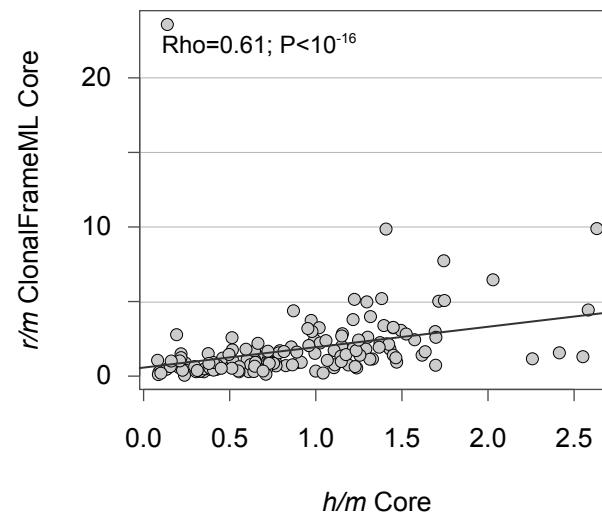

C

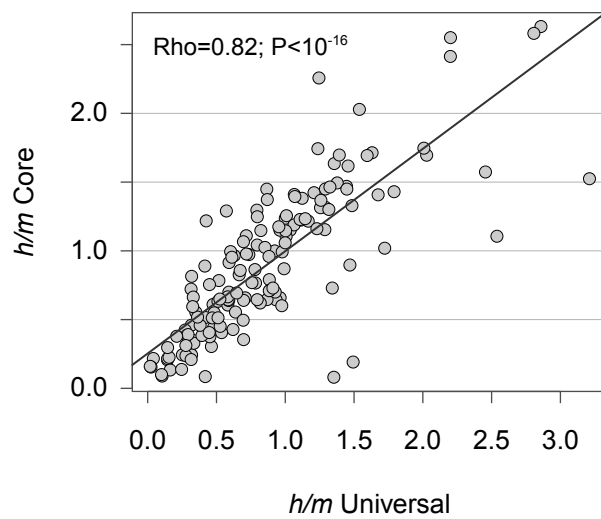

D

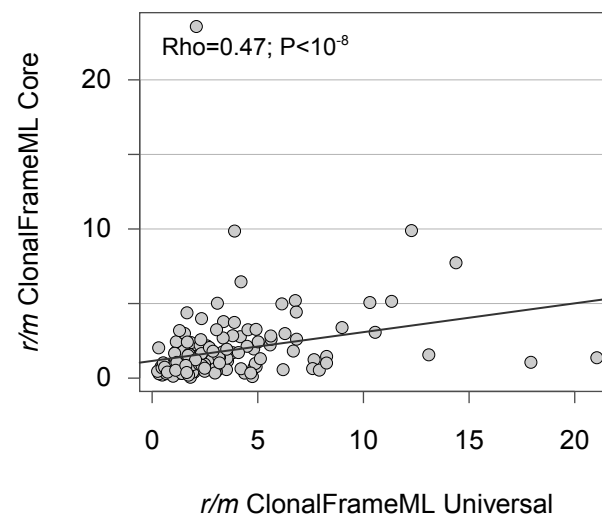

Supplement: Supplementary file 6 — Figure S5. Comparison of recombination detection methods. Recombination rates were estimated based on the ratio of homoplasic to non-homoplasic alleles (h/m) [10] and with ClonalFrameML (r/m) [15]. The two methods were compared on the set of universal genes (A) or on the entire core genome (B) for each species. The performance of each method was then evaluated by comparing the recombination rate on the set of universal genes relative to the complete core genome of each species with h/m ratios (C) and r/m ratios (D). Spearman’s correlation coefficients rho are indicated on top of each graph. (PDF 382 kb) [file 12862_2018_1272_MOESM6_ESM.pdf]

**A**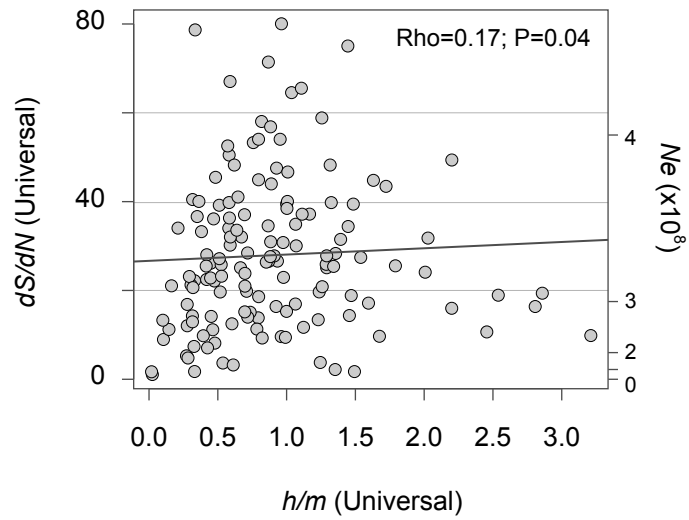**B**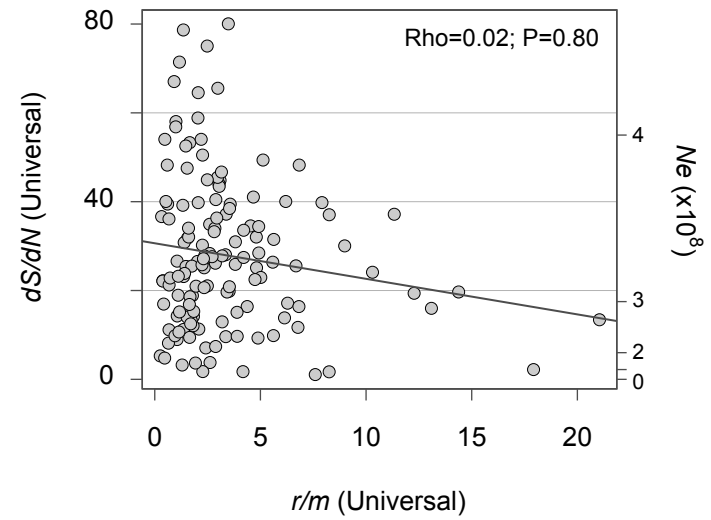**C**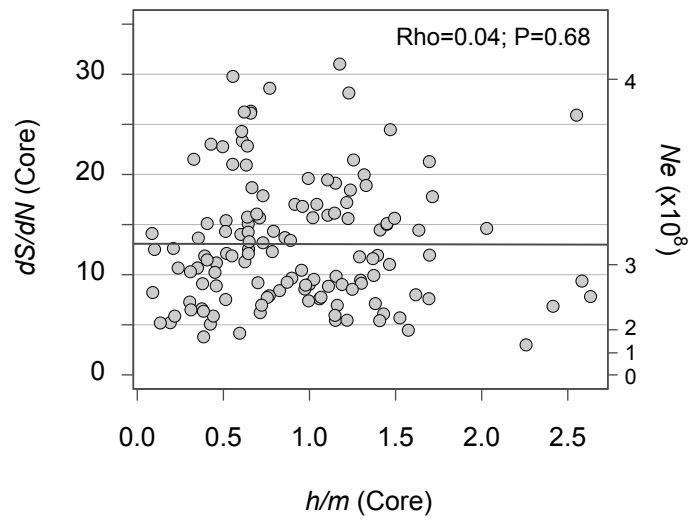**D**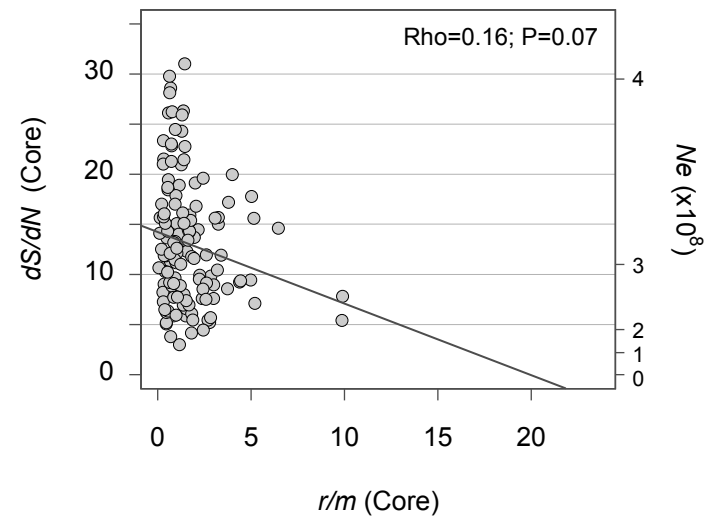

Supplement: Supplementary file 7 — Figure S6. Impact of recombination on estimates of effective population size. Relationship between recombination rate and the effective population size of each species. Recombination rate, estimated from the frequencies of homoplasies, and dS/dN for each species were calculated for universally distributed genes (A) and for the set of core genomes (C), and recombination rate, estimated with ClonalFrameML, and dS/dN for each species were calculated for universally distributed genes (B) and fore the set of core genomes (D). (PDF 359 kb) [file 12862_2018_1272_MOESM7_ESM.pdf]

A

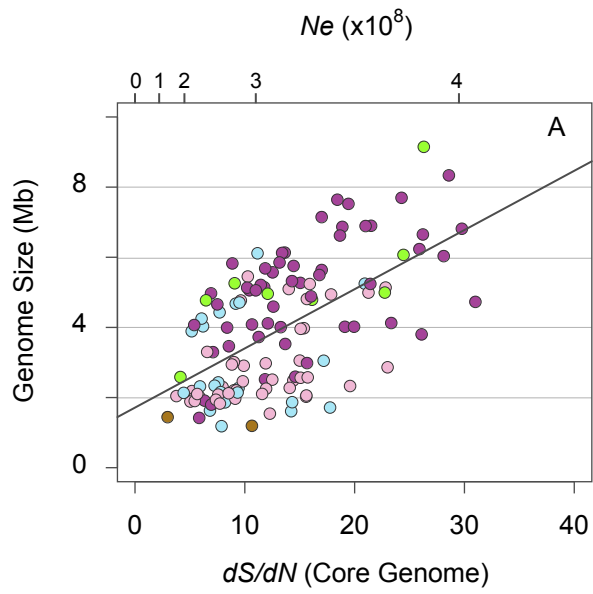

B

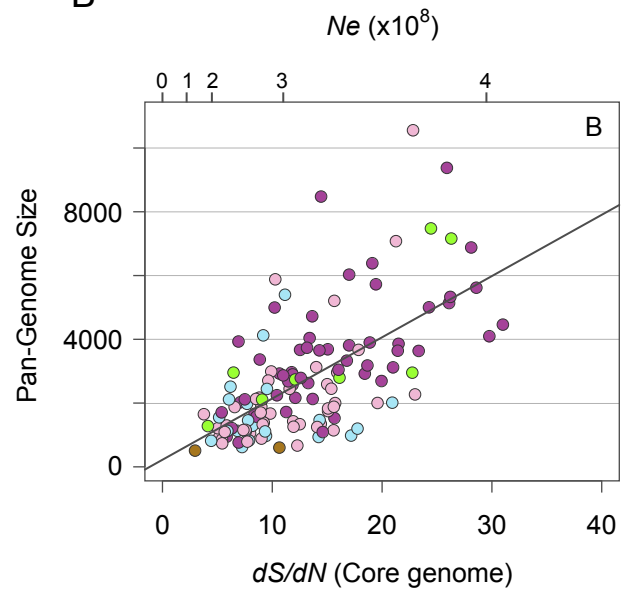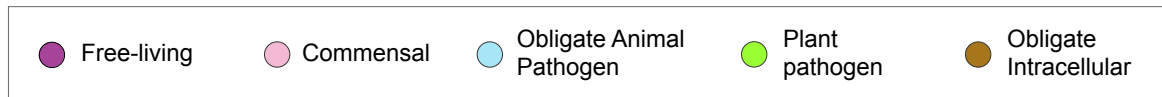

Supplement: Supplementary file 8 — Figure S7. Correlation between genome size, pan-genome size, and effective population sizes as computed from core genomes. Correlation between genome sizes (A) and pan-genomes sizes (Spearman’s rho = 0.32, P < 0.001, PIC correction) (B) when N average dS/dN ratios are determined for core set of genes for each species (Spearman’s rho = 0.48, P < 10− 7, PIC correction). (PDF 240 kb) [file 12862_2018_1272_MOESM8_ESM.pdf]

### Free Living

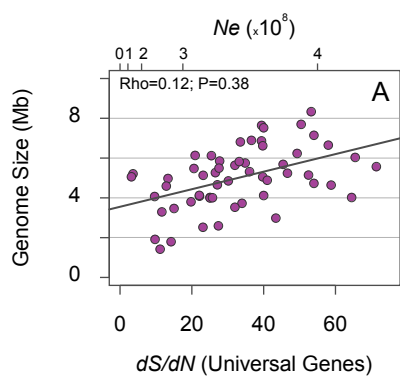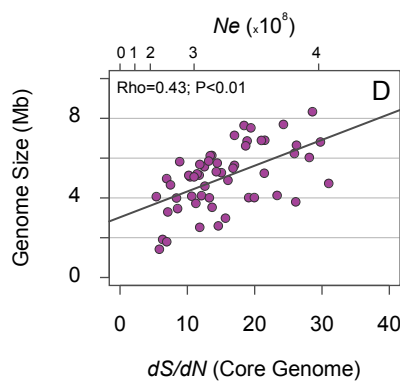

### Commensals

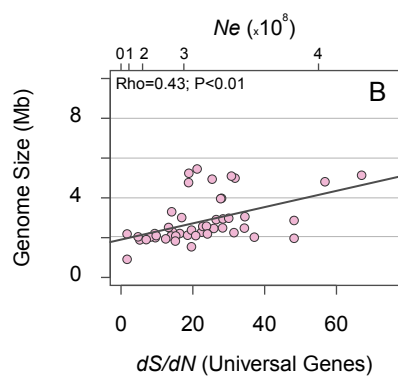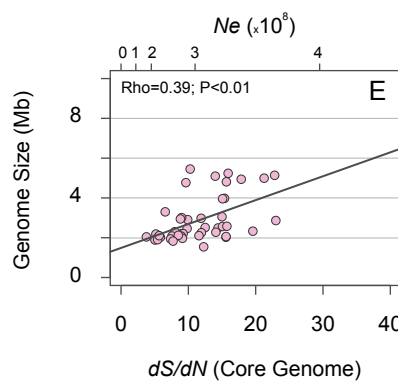

### Obligate Animal Pathogens

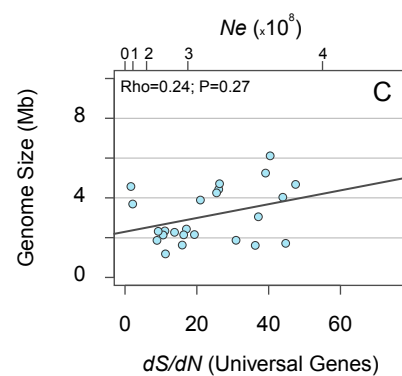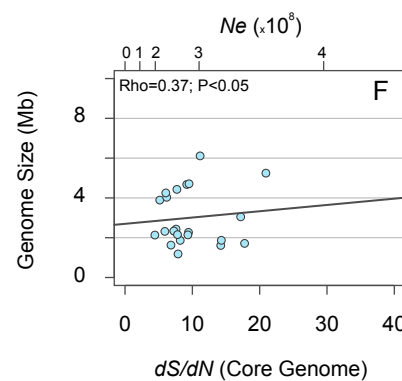

Supplement: Supplementary file 9 — Figure S8. Correlation between genome size and Ne for each lifestyle category. Genome size for a given species was calculated as the average across all sequenced strains. dS/dN ratios were calculated from the common set of universally distributed gene (A–C) and from the core genome of each species (D–F). Spearman’s correlations were adjusted with phylgenetically independent contrasts. (PDF 278 kb) [file 12862_2018_1272_MOESM9_ESM.pdf]

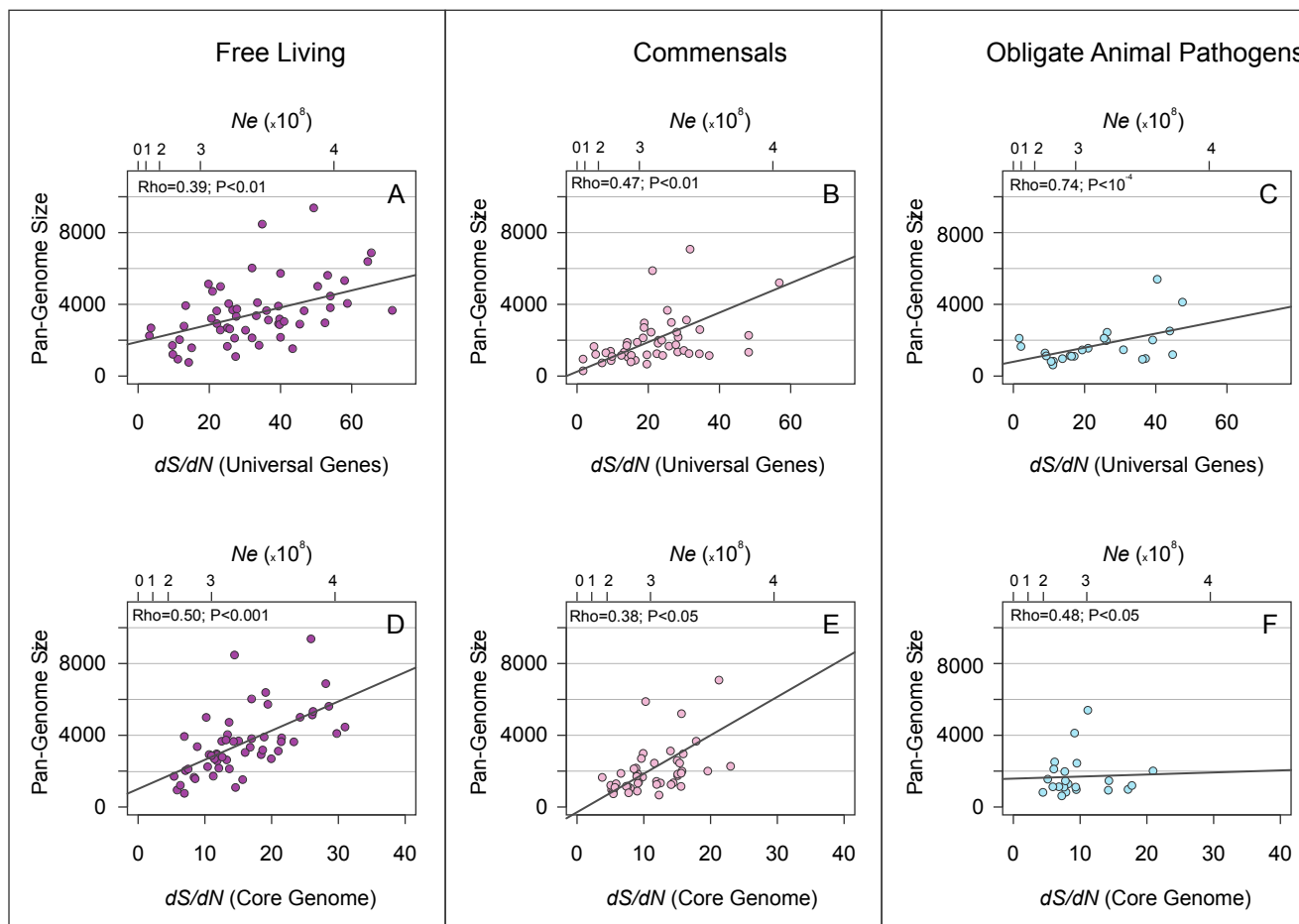

Supplement: Supplementary file 10 — Figure S9. Association between pan-genome size and Ne for each lifestyle category. Pan-genome size for a given species was calculated as the total number of protein families detected normalized by strain number. dS/dN ratios were calculated from the common set of universally distributed gene (A–C) and from the core genome of each species (D–F). Spearman’s correlations were adjusted with phylogenetically independent contrasts. (PDF 274 kb) [file 12862_2018_1272_MOESM10_ESM.pdf]

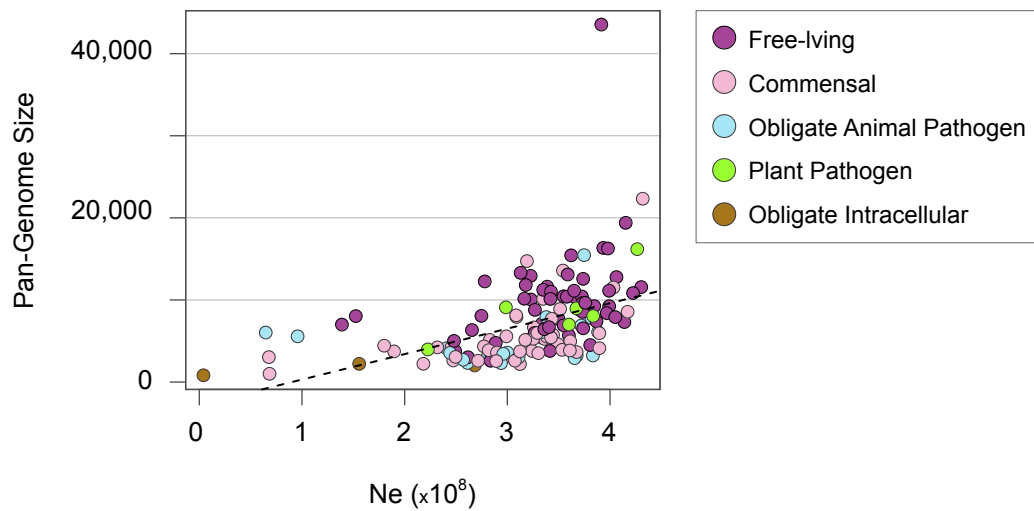

Supplement: Supplementary file 12 — Figure S10. Association between Ne and pan-genome size, adjusted for sample size (Spearman’s rho = 0.48, P < 10− 8, PIC correction). Ne was estimated from dS/dN ratios (Fig. 1). Pan-genome sizes were corrected for sample size by analyzing the same number of genomes for each species while maximizing the divergence rate of the core genome. Using a recursive approach, the pair of the most similar genomes for a species was identified, and one of the two genomes was randomly discarded. This process was repeated until each species was down-sampled to 13 genomes. The pan-genome was then re-built for each species as described above. (PDF 154 kb) [file 12862_2018_1272_MOESM12_ESM.pdf]

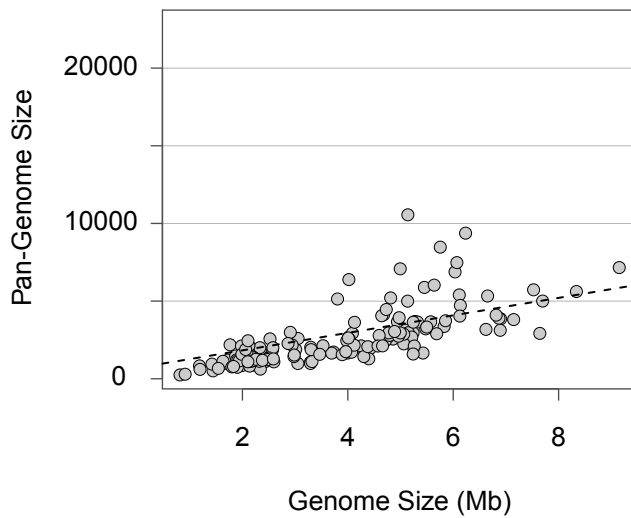

Supplement: Supplementary file 13 — Figure S11. Association between genome size and pan-genome size. Genome sizes represent averages a across all sequenced strains for a given species and pan-genome sizes were calculated as the total number of protein families normalized by the number of strains of a given each species. (PDF 138 kb) [file 12862_2018_1272_MOESM13_ESM.pdf]

Growth rate

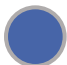

Pan-genome

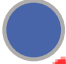

Genome size

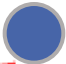

GC-content

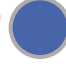

*Ne*

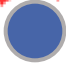

*h/m*

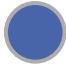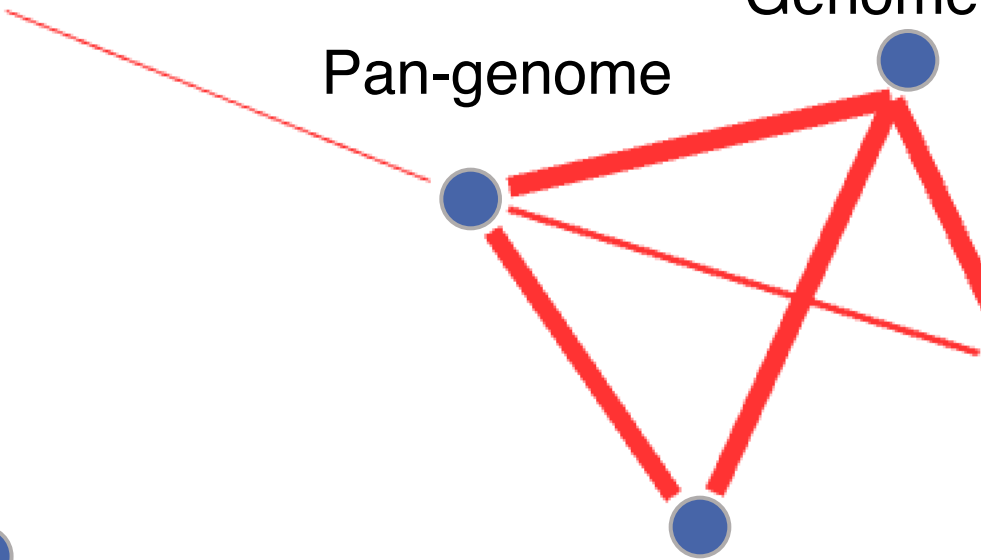

Supplement: Supplementary file 15 — Figure S12. Network of correlations among genomic and lifestyle variables. The correlation network was built using the P-values obtained from the correlation matrix for these quantitative variables (Additional file 14: Table S3). Each node represents a quantitative variable, and the thickness of edges is proportional to the strength of the correlations, defined as –log(P-value). Correlations with P-values > 0.01 were not included in the network. (PDF 112 kb) [file 12862_2018_1272_MOESM15_ESM.pdf]

A

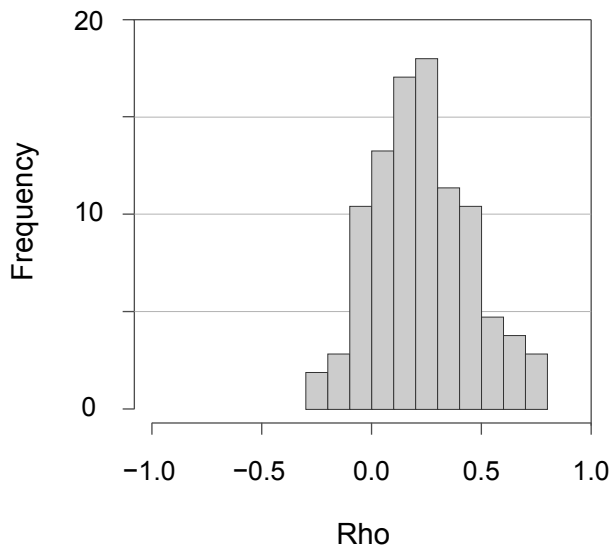

B

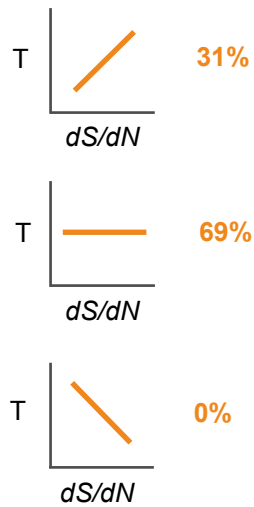

Supplement: Supplementary file 16 — Figure S13. Correlation between gene turnover and effective population size. A. Gene turnover, T, was defined as the rate of gene gains divided by the rate of gene losses on each branch of each species tree. Rates of gene gains and losses were estimated with a posterior probability threshold of 0.3. For each branch of a species trees, the dS/dN ratio was estimated using CodeML (see Methods). The Spearman’s correlation between T and dS/dN ratios was computed for each species, and he distribution of the coefficient rho across species is presented. B. Species were organized into three categories: those with a positive correlation between gene turnover T and dS/dN (top, Spearman’s correlation, P < 0.05); those with no significant correlation between T and dS/dN (middle; Spearman’s correlation, P ≥ 0.05); and those with a negative correlation between T and dS/dN (bottom, Spearman’s correlation, P < 0.05). (PDF 119 kb) [file 12862_2018_1272_MOESM16_ESM.pdf]

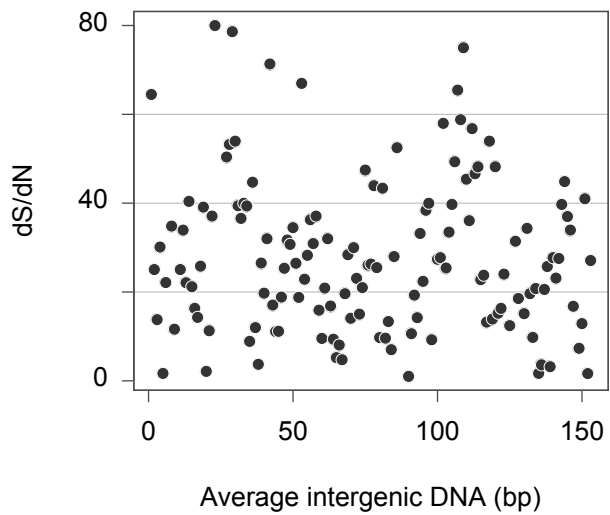

Supplement: Supplementary file 17 — Figure S14. Correlation between dS/dN and intergenic DNA. For each species, dS/dN ratios were estimated as in Fig. 1. Average intergenic DNA of each species corresponds to the average number of base pairs between two consecutive protein-coding genes. No positive correlation was observed between dS/dN and the average intergenic DNA (Spearman’s rho = − 0.22, P < 0.05). (PDF 137 kb) [file 12862_2018_1272_MOESM17_ESM.pdf]
