# Supplementary material for: Factors driving effective population size and pan-genome evolution in bacteria
Source: BMC Evol Biol. 2018 Oct 12;18:153. doi: 10.1186/s12862-018-1272-4 (PMC6186134; doi:10.1186/s12862-018-1272-4)
Supplement: Supplementary file 19 — Table S5. Minimum doubling times of each species and corresponding references. (DOCX 217 kb) [file 12862_2018_1272_MOESM19_ESM.docx]

| **Species** | **Minimal doubling time (h)** | **Reference** |
| --- | --- | --- |
| *Acinetobacter baumannii* | 0.7 | [1] |
| *Acinetobacter pittii* | NA |  |
| *Actinobacillus pleuropneumoniae* | 0.75 | [2] |
| *Aeromonas hydrophila* | 0.35 | [3] |
| *Aggregatibacter actinomycetemcomitans* | 4.9 | [4] |
| *Bacillus amyloliquefaciens* | 8.3 | [5] |
| *Bacillus anthracis* | 0.5 | [6] |
| *Bacillus cereus* | 0.3 | [7] |
| *Bacillus coagulans* | 7.6 | [8] |
| *Bacillus licheniformis* | 0.58 | [9] |
| *Bacillus methylotrophicus* | NA |  |
| *Bacillus pumilus* | 1.9 | [10] |
| *Bacillus subtilis* | 0.43 | [11] |
| *Bacillus thuringiensis* | 0.42 | [12] |
| *Bacteroides fragilis* | 0.63 | [13] |
| *Bifidobacterium bifidum* | 1.5 | [14] |
| *Bifidobacterium breve* | 1.5 | [14] |
| *Bifidobacterium longum* | 1.51 | [15] |
| *Bordetella bronchiseptica* | 1.42 | [16] |
| *Bordetella pertussis* | 3.8 | [17] |
| *Borrelia burgdorferi* | 4 | [18] |
| *Brachyspira hyodysenteriae* | 1 | [19] |
| *Bradyrhizobium japonicum* | 5.7 | [20] |
| *Brucella abortus* | 2 | [21] |
| *Brucella melitensis* | 2 | [22] |
| *Brucella suis* | 2 | [23] |
| *Burkholderia cenocepacia* | 1.9 | [24] |
| *Burkholderia cepacia* | 1.3 | [24] |
| *Burkholderia multivorans* | 1.3 | [24] |
| *Burkholderia pseudomallei* | 1 | [25] |
| *Burkholderia stagnalis* | NA |  |
| *Burkholderia territorii* | NA |  |
| *Burkholderia ubonensis* | NA |  |
| *Burkholderia vietnamiensis* | 2.1 | [24] |
| *Campylobacter fetus* | NA |  |
| *Campylobacter jejuni* | 1.5 | [26] |
| *Chlamydia psittaci* | 2 | [27] |
| *Chlamydia trachomatis* | 24 | [28] |
| *Citrobacter freundii* | 0.8 | [29] |
| *Clostridium botulinum* | 1 | [30] |
| *Clostridium perfringens* | 0.2 | [31] |
| *Comamonas testosteroni* | 1.2 | [32] |
| *Corynebacterium diphtheriae* | 1 | microbewiki |
| *Corynebacterium pseudotuberculosis* | NA |  |
| *Dehalococcoides mccartyi* | 19.2 | [25] |
| *Enterobacter aerogenes* | 0.4 | [33] |
| *Enterobacter asburiae* | NA |  |
| *Enterobacter cloacae* | 0.5 | [34] |
| *Enterobacter hormaechei* | NA |  |
| *Enterococcus faecalis* | 0.5 | [35] |
| *Enterococcus faecium* | 0.7 | [36] |
| *Escherichia albertii* | NA |  |
| *Escherichia coli* | 0.35 | [37] |
| *Fusobacterium nucleatum* | 0.72 | [38] |
| *Gallibacterium anatis* | 0.7 | [39] |
| *Gardnerella vaginalis* | NA |  |
| *Haemophilus influenzae* | 0.5 | [37] |
| *Haemophilus parainfluenzae* | 0.6 | [40] |
| *Helicobacter pylori* | 2.4 | [41] |
| *Kingella kingae* | NA |  |
| *Klebsiella oxytoca* | 0.5 | [42] |
| *Klebsiella pneumoniae* | 0.6 | [43] |
| *Lactobacillus casei* | 1.2 | [44] |
| *Lactobacillus crispatus* | 1.7 | [45] |
| *Lactobacillus delbrueckii* | 0.7 | [46] |
| *Lactobacillus fermentum* | 0.8 | [47] |
| *Lactobacillus helveticus* | 1 | [48] |
| *Lactobacillus kunkeei* | 1 | [49] |
| *Lactobacillus paracasei* | 0.9 | [50] |
| *Lactobacillus plantarum* | 1.6 | [51] |
| *Lactobacillus rhamnosus* | 0.4 | [52] |
| *Lactococcus lactis* | 0.7 | [53] |
| *Legionella pneumophila* | 3.3 | [54] |
| *Leptospira borgpetersenii* | NA |  |
| *Leptospira interrogans* | 6 | [55] |
| *Leptospira kirschneri* | 6 | [56] |
| *Leptospira noguchii* | 6 | http://www.leptospirosis.org |
| *Leptospira santarosai* | 6 | http://www.leptospirosis.org |
| *Leptospira weilii* | 6 | http://www.leptospirosis.org |
| *Leuconostoc mesenteroides* | 4.7 | [57] |
| *Listeria monocytogenes* | 1 | [37] |
| *Methanosarcina mazei* | 16.7 | [58] |
| *Microcystis aeruginosa* | 1.2 | [59] |
| *Moraxella catarrhalis* | NA |  |
| *Morganella morganii* | NA |  |
| *Mycobacterium abscessus* | 4 | [60] |
| *Mycobacterium africanum* | 24 | [61] |
| *Mycobacterium bovis* | 23 | [62] |
| *Mycobacterium tuberculosis* | 19 | [62] |
| *Mycoplasma pneumoniae* | 6 | [63] |
| *Neisseria gonorrhoeae* | 0.58 | [64] |
| *Neisseria meningitidis* | 0.72 | [65] |
| *Oenococcus oeni* | 8.7 | [66] |
| *Paenibacillus polymyxa* | 0.8 | [67] |
| *Pasteurella multocida* | 1 | [68] |
| *Pectobacterium carotovorum* | NA |  |
| *Peptoclostridium difficile* | 0.3 | [69] |
| *Porphyromonas gingivalis* | 2.7 | [70] |
| *Prochlorococcus marinus* | 17 | [71] |
| *Propionibacterium freudenreichii* | 10.3 | [72] |
| *Proteus mirabilis* | 0.4 | [73] |
| *Pseudomonas aeruginosa* | 0.5 | [37] |
| *Pseudomonas amygdali* | NA |  |
| *Pseudomonas chlororaphis* | 2 | [74] |
| *Pseudomonas denitrificans* | 0.9 | [75] |
| *Pseudomonas fluorescens* | 0.9 | [76] |
| *Pseudomonas putida* | 1.1 | [77] |
| *Pseudomonas stutzeri* | 1.9 | [78] |
| *Pseudomonas syringae* | 1.47 | [79] |
| *Salinispora arenicola* | NA |  |
| *Salinispora pacifica* | NA |  |
| *Salmonella enterica* | 0.3 | [80] |
| *Serratia marcescens* | 0.7 | [81] |
| *Staphylococcus aureus* | 0.4 | [82] |
| *Staphylococcus epidermidis* | 0.8 | [83] |
| *Staphylococcus haemolyticus* | 1 | [84] |
| *Staphylococcus warneri* | NA |  |
| *Stenotrophomonas maltophilia* | 0.3 | [85] |
| *Streptococcus agalactiae* | 1.8 | [86] |
| *Streptococcus anginosus* | 2.5 | [87] |
| *Streptococcus dysgalactiae* | NA |  |
| *Streptococcus equi* | 1.5 | [88] |
| *Streptococcus gordonii* | 1 | [89] |
| *Streptococcus mitis* | 0.8 | [90] |
| *Streptococcus mutans* | 0.75 | [91] |
| *Streptococcus oralis* | 1.1 | [92] |
| *Streptococcus parasanguinis* | 0.8 | [93] |
| *Streptococcus pneumoniae* | 0.5 | [94] |
| *Streptococcus pseudopneumoniae* | NA |  |
| *Streptococcus pyogenes* | 0.4 | [95] |
| *Streptococcus salivarius* | 0.5 | [96] |
| *Streptococcus sanguinis* | 1.4 | [97] |
| *Streptococcus sobrinus* | 1 | [98] |
| *Streptococcus suis* | 0.8 | [99] |
| *Tropheryma whipplei* | 28 | [100] |
| *Vibrio alginolyticus* | 0.2 | [101] |
| *Vibrio campbellii* | NA |  |
| *Vibrio cholerae* | 0.2 | [28] |
| *Vibrio cyclitrophicus* | NA |  |
| *Vibrio harveyi* | 1 | [102] |
| *Vibrio parahaemolyticus* | 0.2 | [103] |
| *Vibrio splendidus* | NA |  |
| *Vibrio vulnificus* | 0.16 | [28] |
| *Xanthomonas axonopodis* | 7 | [104] |
| *Xanthomonas campestris* | 3 | [105] |
| *Xanthomonas citri* | 2 | [106] |
| *Xanthomonas oryzae* | 2 | [107] |
| *Xanthomonas perforans* | NA |  |
| *Xylella fastidiosa* | 5.13 | [108] |
| *Yersinia enterocolitica* | 0.5 | [109] |
| *Yersinia frederiksenii* | NA |  |
| *Yersinia pestis* | 1.25 | [25] |
| *Yersinia pseudotuberculosis* | 0.5 | [110] |

References

1. Antunes LC, Imperi F, Carattoli A, Visca P. Deciphering the multifactorial nature of Acinetobacter baumannii pathogenicity. PLoS One. 2011;6:e22674.

2. Fuller TE, Shea RJ, Thacker BJ, Mulks MH. Identification of in vivo induced genes in Actinobacillus pleuropneumoniae. Microb Pathog. 1999;27:311-27.

3. Hudson JA. Effect of pre-incubation temperature on the lag time of Aeromonas hydrophila. Lett Appl Microbiol. 1993;16:274-276.

4. Cheng YA, Jee J, Hsu G, Huang Y, Chen C, et al. A markerless protocol for genetic analysis of Aggregatibacter actinomycetemcomitans. J Formos Med Assoc. 2014;113:114-23.

5. Abd-Elhalem BT, El-Sawy M, Gamal RF, Abou-Taleb KA. Production of amylases from Bacillus amyloliquefaciens under submerged fermentation using some agro-industrial by-products. Ann Agric Sci. 2015;60:193-202.

6. Chakrabarty K, Wu W, Booth JL, Duggan ES, Coggeshall KM, et al. Bacillus anthracis spores stimulate cytokine and chemokine innate immune responses in human alveolar macrophages through multiple mitogen-activated protein kinase pathways. Infect Immun. 2006;74:4430-8.

7. Olmez HK, Aran N. Modeling the growth kinetics of Bacillus cereus as a function of temperature, pH, sodium lactate and sodium chloride concentrations. Int J Food Microbiol. 2005;98:135-43.

8. Uma B, Sandhya S. Pyridine degradation and heterocyclic nitrification by Bacillus coagulans. Can J Microbiol. 1997;43:595-8.

9. van Dijk-Salkinoja MS, Planta RJ. Rate of ribosome production in Bacillus licheniformis. J Bacteriol. 1971;105:20-7.

10. Molina CA, Cana-Roca JF, Osuna A, Vilchez S. Selection of a Bacillus pumilus strain highly active against Ceratitis capitata (Wiedemann) larvae. Appl Environ Microbiol. 2010;76:1320-7.

11. Yoshikawa H, O'Sullivan A, Sueoka N. Sequential replication of the Bacillus subtilis chromosome. 3. Regulation of initiation. Proc Natl Acad Sci U S A. 1964;52: 973-80.

12. Kashyap S, Amla DV. Characterisation of Bacillus thuringiensis kurstaki strains by toxicity, plasmid profiles and numerical analysis of their cryIA genes. Afr J Biotechnol. 2007;6.

13. Sturr MG, Guffanti AA, Krulwich TA. Growth and bioenergetics of alkaliphilic Bacillus firmus OF4 in continuous culture at high pH. J Bacteriol. 1994;176:3111-6.

14. Dubey UK, Mistry VV. Growth characteristics of bifidobacteria in infant formulas. J Dairy Sci. 1996;79:1146-55.

15. Garro MS, Aguirre L, Savoy de Giori G. Biological activity of Bifidobacterium longum in response to environmental pH. Appl Microbiol Biotechnol. 2006;70:612-7.

16. Yuk MH, Harvill ET, Miller JF. The BvgAS virulence control system regulates type III secretion in Bordetella bronchiseptica. Mol Microbiol. 1998;28:945-59.

17. Frohlich BT, De Bernardez Clark ER, Siber GR, Swartz RW. Improved pertussis toxin production by Bordetella pertussis through adjusting the growth medium's ionic composition. J Biotechnol. 1995;39:205-19.

18. De Silva AM, Fikrig E. Growth and migration of Borrelia burgdorferi in Ixodes ticks during blood feeding. Am J Trop Med Hyg. 1995;53:397-404.

19. Paster BJ. Phylum XV Spirochaetes. In: Krieg NR, Ludwig W, Whitman WB, Hedlund BP, Paster BJ et al., editors. Bergey’s Manual of Systematic Bacteriology. New-York NY: Springer-Verlag. 2010.

20. Miclea PS, Peter M, Vegh G, Cinege G, Kiss E, et al. Atypical transcriptional regulation and role of a new toxin-antitoxin-like module and its effect on the lipid composition of Bradyrhizobium japonicum. Mol Plant Microbe Interact. 2010;23:638-50.

21. Hoover DL, Friedlander AM. Brucellosis. In: Zajtchuk R, editor. Textbook of Military Medicine: Medical Aspects of Chemical and Biological Warfare: The Virtual Naval Hospital Project. Bethesda, Maryland: Office of The Surgeon General. Department of the Army, United States of America. 2005; pp513-21.

22. Gallot-Lavallee T, Zygmunt MS, Cloeckaert A, Bezard G, Dubray G. Growth phase-dependent variations in the outer membrane protein profile of Brucella melitensis. Res Microbiol. 1995;146: 227-36.

23. Ekaza E, Guilloteau L, Teyssier J, Liautard JP, Kohler S. Functional analysis of the ClpATPase ClpA of Brucella suis, and persistence of a knockout mutant in BALB/c mice. Microbiology. 2000;146:1605-16.

24. Caraher E, Duff C, Mullen T, Mc Keon S, Murphy P, et al. Invasion and biofilm formation of Burkholderia dolosa is comparable with Burkholderia cenocepacia and Burkholderia multivorans. J Cyst Fibros. 2007;6:49-56.

25. Vieira-Silva S, Rocha EP. The systemic imprint of growth and its uses in ecological (meta)genomics. PLoS Genet. 2010;6:e1000808.

26. Rollins DM, Coolbaugh JC, Walker RI, Weiss E. Biphasic culture system for rapid Campylobacter cultivation. Appl Environ Microbiol. 1983;45:284-9.

27. Alexander JJ. Effect of infection with the meningopneumonitis agent on deoxyribonucleic acid and protein synthesis by its L-cell host. J Bacteriol. 1969;97:653-7.

28. Holt JG. Archaeobacteria, Cyanobacteria, and remaining Gram-negative Bacteria. In: Williams ST, Sharpe ME, Holt JG, editors. Bergey's Manual of Systematic Bacteriology. Baltimore: Williams and Wilkins. 1989.

29. Sawai T, Nakajima S, Morohoshi T, Yamagishi S. Thermolabile repression of cephalosporinase synthesis in Citrobacter freundii. Microbiol Immunol. 1977;21:631-8.

30. Stringer SC, Webb MD, George SM, Pin C, Peck MW. Heterogeneity of times required for germination and outgrowth from single spores of nonproteolytic Clostridium botulinum. Appl Environ Microbiol. 2005;71:4998-5003.

31. Bryant AE, Stevens DL. The pathogenesis of gas gangrene. In: Rood JI, McClane BA, Songer JG, Titball RW, editors. The Clostridia: Molecular Biology and Pathogen- esis. San Diego CA: Academic Press. 1997. pp185-96.

32. Pruneda-Paz JL, Linares M, Cabrera JE, Genti-Raimondi S (2004) TeiR, a LuxR-type transcription factor required for testosterone degradation in Comamonas testosteroni. J Bacteriol. 2004;186:1430-7.

33. Ingham CJ, van den Ende M, Wever PC, Schneeberger PM. Rapid antibiotic sensitivity testing and trimethoprim-mediated filamentation of clinical isolates of the Enterobacteriaceae assayed on a novel porous culture support. J Med Microbiol. 2006;55:1511-9.

34. Kelly CD, Rahn O. The growth rate of individual bacterial cells. J Bacteriol. 1932;23:147-153.

35. Sarantinopoulos P, Makras L, Vaningelgem F, Kalantzopoulos G, De Vuyst L, et al. Growth and energy generation by Enterococcus faecium FAIR-E 198 during citrate metabolism. Int J Food Microbiol. 2003;84:197-206.

36. Lam MM, Seemann T, Tobias NJ, Chen H, Haring V, et al. Comparative analysis of the complete genome of an epidemic hospital sequence type 203 clone of vancomycin-resistant Enterococcus faecium. BMC Genomics. 2013;14:595.

37. Rubin LG. Comparison of in vivo and in vitro multiplication rates of Haemophilus influenzae type b. Infect Immun. 1986;52:911-3.

38. Mangels JI, Lindberg LH, Vosti KL. Quantitative evaluation of three commercial blood culture media for growth of anaerobic organisms. J Clin Microbiol. 1978;7:59-62.

39. Bager RJ, Persson G, Nesta B, Soriani M, Serino L, et al. Outer membrane vesicles reflect environmental cues in Gallibacterium anatis. Vet Microbiol. 2013;167:565-72.

40. Artman M, Domenech E, Weiner M. Growth of Haemophilus influenzae in simulated blood cultures supplemented with hemin and NAD. J Clin Microbiol. 1983;18:376-9.

41. Vega AE, Cortinas TI, Mattana CM, Silva HJ, Puig De Centorbi O. Growth of Helicobacter pylori in medium supplemented with cyanobacterial extract. J Clin Microbiol. 2003;41:5384-8.

42. Guimarães JR, Farah CTR, Fadini PS. Short-term toxicity test: Monitoring Klebsiella oxytoca bacterium respiration using a flow injection analysis/conductometric system. J Braz Chem Soc. 2012;23:461-7.

43. Regue M, Hita B, Pique N, Izquierdo L, Merino S, et al. A gene, uge, is essential for Klebsiella pneumoniae virulence. Infect Immun. 2004;72:54-61.

44. Lee YK, Ho PS, Low CS, Arvilommi H, Salminen S. Permanent colonization by Lactobacillus casei is hindered by the low rate of cell division in mouse gut. Appl Environ Microbiol. 2004;70:670-4.

45. Trojok R. Towards the utilization of lactic acid bacteria as contraceptive agents Project title: "The liquid condom project". Berlin: University of Freiburg. 2013.

46. Riazi A, Ziar H. Growth and viability of yogurt starter organisms in honey-sweetened skimmed milk. Afr J Biotechnol. 2008;7:2055-63.

47. Kalsum U, Sjofjan O. The growth of Lactobacillus fermentum isolated from quail intestine on rice bran medium. J Trop Life Sci. 2012;2:58-61.

48. Neviani E, Carminati D, Veaux M, Hermier J, Giraffa G. Characterization of Lactobacillus helveticus strains resistant to lysozyme. Lait. 1991;71:65-73.

49. Tamarit D, Ellegaard KM, Wikander J, Olofsson T, Vasquez A, et al. Functionally structured genomes in Lactobacillus kunkeei colonizing the honey crop and food products of honeybees and stingless bees. Genome Biol Evol. 2015;7:1455-73.

50. Budinich MF, Perez-Diaz I, Cai H, Rankin SA, Broadbent JR, et al. Growth of Lactobacillus paracasei ATCC 334 in a cheese model system: a biochemical approach. J Dairy Sci. 2011;94:5263-77.

51. Barcena JM, Sineriz F, Gonzalez de Llano D, Rodriguez A, Suarez JE (1998) Chemostat production of plantaricin C by Lactobacillus plantarum LL441. Appl Environ Microbiol. 1998;64:3512-4.

52. Valik L, Medveďová A, Liptakova D. Characterization of the growth of Lactobacillus rhamnosus GG in milk at suboptimal temperatures. J Food Nutr Res. 2008;47:60-7.

53. Andersen HW, Solem C, Hammer K, Jensen PR. Twofold reduction of phosphofructokinase activity in Lactococcus lactis results in strong decreases in growth rate and in glycolytic flux. J Bacteriol. 2001;183:3458-67.

54. Saito A, Rolfe RD, Edelstein PH, Finegold SM. Comparison of liquid growth media for Legionella pneumophila. J Clin Microbiol. 1981;14:623-7.

55. Mohammed H, Nozha C, Hakim K, Abdelaziz A, Rekia R. Leptospira: Morphology, classification and pathogenesis. J Bacteriol Parasitol. 2011;2:120.

56. Adler B, de la Pena Moctezuma A. Leptospira and leptospirosis. Vet Microbiol. 2010;140:287-96.

57. Hamasaki Y, Ayaki M, Fuchu H, Sugiyama M, Morita H. Behavior of psychrotrophic lactic acid bacteria isolated from spoiling cooked meat products. Appl Environ Microbiol. 2003;69:3668-71.

58. Mah RA. Isolation and characterization of Methanococcus mazei. Curr Microbiol. 1980;3:321-6.

59. Wilson AE, Wilson WA, Hay ME. Intraspecific variation in growth and morphology of the bloom-forming cyanobacterium Microcystis aeruginosa. Appl Environ Microbiol. 2006;72:7386-9.

60. Cortes MA, Nessar R, Singh AK. Laboratory maintenance of Mycobacterium abscessus. Curr Protoc Microbiol. 2010. Chapter 10: Unit 10D 11.

61. Gehre F, Otu J, DeRiemer K, de Sessions PF, Hibberd ML, et al. Deciphering the growth behaviour of Mycobacterium africanum. PLoS Negl Trop Dis. 2013;7:e2220.

62. Dunn PL, North RJ. Virulence ranking of some Mycobacterium tuberculosis and Mycobacterium bovis strains according to their ability to multiply in the lungs, induce lung pathology, and cause mortality in mice. Infect Immun. 1995;63:3428-37.

63. Peterson SN, Fraser CM. The complexity of simplicity. Genome Biol. 2001;2: COMMENT2002.

64. Hopper S, Vasquez B, Merz A, Clary S, Wilbur JS, et al. Effects of the immunoglobulin A1 protease on Neisseria gonorrhoeae trafficking across polarized T84 epithelial monolayers. Infect Immun. 2000;68:906-11.

65. Deeudom M, Koomey M, Moir JW. Roles of c-type cytochromes in respiration in Neisseria meningitidis. Microbiology. 2008;154:2857-64.

66. Maicas S, González-cabo P, Ferrer S, Pardo I. Production of Oenococcus oeni biomass to induce malolactic fermentation in wine by control of pH and substrate addition. Biotechnol Lett. 1999;21:349-53.

67. Bosshard PP, Zbinden R, Altwegg M. Paenibacillus turicensis sp. nov., a novel bacterium harbouring heterogeneities between 16S rRNA genes. Int J Syst Evol Microbiol. 2002;52:2241-9.

68. Xia X, Wei T, Xie Z, Danchin A. Genomic changes in nucleotide and dinucleotide frequencies in Pasteurella multocida cultured under high temperature. Genetics. 2002;161:1385-94.

69. Lancaster WA, Utturkar SM, Poole FL, Klingeman DM, Elias DA, et al. Near-complete genome sequence of Clostridium paradoxum strain JW-YL-7. Genome Announc. 2016;4.

70. Marsh PD, McDermid AS, McKee AS, Baskerville A. The effect of growth rate and haemin on the virulence and proteolytic activity of Porphyromonas gingivalis W50. Microbiology. 1994;140:861-5.

71. Shalapyonok A, Olson RJ, Shalapyonok LS. Ultradian growth in Prochlorococcus spp. Appl Environ Microbiol. 1998;64:1066-9.

72. Gagnaire V, Jardin J, Rabah H, Briard-Bion V, Jan G. Emmental cheese environment enhances Propionibacterium freudenreichii stress tolerance. PLoS One. 2015;10:e0135780.

73. Sosa V, Schlapp G, Zunino P. Proteus mirabilis isolates of different origins do not show correlation with virulence attributes and can colonize the urinary tract of mice. Microbiology. 2006;152: 2149-57.

74. Fukui R, Schroth MN, Hendson M, Hancock JG. Interaction between strains of pseudomonads in sugar beet spermospheres and their relationship to pericarp colonization by Pythium ultimum in soil. Phytopathology. 1994;84:1322-30.

75. Arasu MV, Sarkar R, Sekar BS, Kumar V, Rathnasingh C, et al. Isolation of a novel Pseudomonas species SP2 producing vitamin B12 under aerobic condition. Biotechnol Bioprocess Eng. 2013;18:43-51.

76. O'Mahony FC, Papkovsky DB. Rapid high-throughput assessment of aerobic bacteria in complex samples by fluorescence-based oxygen respirometry. Appl Environ Microbiol. 2006;72:1279-87.

77. Ahn IS, Ghiorse WC, Lion LW, Shuler ML. Growth kinetics of Pseudomonas putida G7 on naphthalene and occurrence of naphthalene toxicity during nutrient deprivation. Biotechnol Bioeng. 1998;59:587-94.

78. Carlson CA, Ingraham JL. Comparison of denitrification by Pseudomonas stutzeri, Pseudomonas aeruginosa, and Paracoccus denitrificans. Appl Environ Microbiol. 1983;45:1247-53.

79. Keith LM, Partridge JE, Bender CL. dnaK and the heat stress response of Pseudomonas syringae pv. glycinea. Mol Plant Microbe Interact. 1999;12:563-74.

80. Molloy S. Salmonella's exit strategy. Nat Rev Microbiol. 2010;8:839.

81. Fedrigo GV, Campoy EM, Di Venanzio G, Colombo MI, Garcia Vescovi E. Serratia marcescens is able to survive and proliferate in autophagic-like vacuoles inside non-phagocytic cells. PLoS One. 2011;6:e24054.

82. Somerville GA, Said-Salim B, Wickman JM, Raffel SJ, Kreiswirth BN, et al. Correlation of acetate catabolism and growth yield in Staphylococcus aureus: implications for host-pathogen interactions. Infect Immun. 2003;71:4724-32.

83. Gottenbos B, van der Mei HC, Busscher HJ. Initial adhesion and surface growth of Staphylococcus epidermidis and Pseudomonas aeruginosa on biomedical polymers. J Biomed Mater Res. 2000;50:208-14.

84. Cerca N, Martins S, Sillankorva S, Jefferson KK, Pier GB, et al. Effects of growth in the presence of subinhibitory concentrations of dicloxacillin on Staphylococcus epidermidis and Staphylococcus haemolyticus biofilms. Appl Environ Microbiol. 2005;71:8677-82.

85. Mahdi O, Eklund B, Fisher N. Laboratory culture and maintenance of Stenotrophomonas maltophilia. Curr Protoc Microbiol. 2014;32:Unit 6F1.

86. Malin G, Paoletti LC. Use of a dynamic in vitro attachment and invasion system (DIVAS) to determine influence of growth rate on invasion of respiratory epithelial cells by group B Streptococcus. Proc Natl Acad Sci U S A. 2001;98:13335-40.

87. de Jong MH, van der Hoeven JS, van OJ, Olijve JH. Growth of oral Streptococcus species and Actinomyces viscosus in human saliva. Appl Environ Microbiol. 1984;47:901-4.

88. Meehan M, Burke FM, Macken S, Owen P. Characterization of the haem-uptake system of the equine pathogen Streptococcus equi subsp. equi. Microbiology. 2010;156:1824-35.

89. Zeng L, Martino NC, Burne RA. Two gene clusters coordinate galactose and lactose metabolism in Streptococcus gordonii. Appl Environ Microbiol. 2012;78: 5597-605.

90. Fathilah AR, Rahim ZH, Othman Y, Yusoff M. Bacteriostatic effect of Piper betle and Psidium guajava extracts on dental plaque bacteria. Pak J Biol Sci. 2009;12:518-21.

91. Shimamoto T, Fukui K, Kodama T, Shimono T, Ohta H, et al. Effects of oxygen on growth of Streptococcus mutans. Shika Kiso Igakkai Zasshi. 1990.32:10-9.

92. Wilkins JC, Homer KA, Beighton D. Altered protein expression of Streptococcus oralis cultured at low pH revealed by two-dimensional gel electrophoresis. Appl Environ Microbiol. 2001;67:3396-405.

93. Chen YY, Shieh HR, Chang YC. The expression of the fim operon is crucial for the survival of Streptococcus parasanguinis FW213 within macrophages but not acid tolerance. PLoS One. 2013;8:e66163.

94. Small PM, Tauber MG, Hackbarth CJ, Sande MA. Influence of body temperature on bacterial growth rates in experimental pneumococcal meningitis in rabbits. Infect Immun. 1986:52:484-7.

95. Biswas I, Germon P, McDade K, Scott JR (2001) Generation and surface localization of intact M protein in Streptococcus pyogenes are dependent on sagA. Infect Immun. 2001;69:7029-38.

96. Thomas S, Brochu D, Vadeboncoeur C. Diversity of Streptococcus salivarius ptsH mutants that can be isolated in the presence of 2-deoxyglucose and galactose and characterization of two mutants synthesizing reduced levels of HPr, a phosphocarrier of the phosphoenolpyruvate:sugar phosphotransferase system. J Bacteriol. 2001;183: 5145-54.

97. Wen ZT, Yates D, Ahn SJ, Burne RA. Biofilm formation and virulence expression by Streptococcus mutans are altered when grown in dual-species model. BMC Microbiol. 2010;10:111.

98. Homer KA, Patel R, Beighton D. Effects of N-acetylglucosamine on carbohydrate fermentation by Streptococcus mutans NCTC 10449 and Streptococcus sobrinus SL-1. Infect Immun. 1993;61:295-302.

99. Wu Z, Wang W, Tang M, Shao J, Dai C, et al. Comparative genomic analysis shows that Streptococcus suis meningitis isolate SC070731 contains a unique 105K genomic island. Gene. 2014;535:156-64.

100. Renesto P, Crapoulet N, Ogata H, La Scola B, Vestris G, et al. Genome-based design of a cell-free culture medium for Tropheryma whipplei. Lancet. 2003;362:447-9.

101. Ulitzur S. Vibrio parahaemolyticus and Vibrio alginolyticus: Short generation-time marine bacteria. Microb Ecol. 1974;1:127-35.

102. Anetzberger C, Pirch T, Jung K. Heterogeneity in quorum sensing-regulated bioluminescence of Vibrio harveyi. Mol Microbiol. 2009;73:267-77.

103. Twedt RM, Novelli RM. Modified selective and differential isolation medium for Vibrio parahaemolyticus. Appl Microbiol. 1971;22:593-9.

104. Wichmann G, Ritchie D, Kousik CS, Bergelson J. Reduced genetic variation occurs among genes of the highly clonal plant pathogen Xanthomonas axonopodis pv. vesicatoria, including the effector gene avrBs2. Appl Environ Microbiol. 2005;71: 2418-32.

105. Pena C, Galindo E, Diaz M. Effectiveness factor in biological external convection: study in high viscosity systems. J Biotechnol. 2002;95:1-12.

106. Silva IC, Regasini LO, Petronio MS, Silva DH, Bolzani VS, et al. Antibacterial activity of alkyl gallates against Xanthomonas citri subsp. citri. J Bacteriol. 2013;195:85-94.

107. Ehrlich M, Lin FH, Ehrlich K, Brown SL, Mayo JA. Changes in macromolecular synthesis in Xanthomonas oryzae infected with bacteriophage XP-12. J Virol. 1977;23:517-23.

108. Feil H, Purcell AH. Temperature-dependent growth and survival of Xylella fastidiosa in vitro and in potted grapevines. Pl Dis. 2001;85:1230-4.

109. Neuhaus K, Rapposch S, Francis KP, Scherer S. Restart of exponential growth of cold-shocked Yersinia enterocolitica occurs after down-regulation of cspA1/A2 mRNA. J Bacteriol. 2000;182:3285-8.

110. Dreyfus LA, Brubaker RR. Consequences of aspartase deficiency in Yersinia pestis. J Bacteriol. 1978;136:757-64.
